# Drug treatment effects on outcomes in heart failure with preserved ejection fraction: a systematic review and meta-analysis

**DOI:** 10.1136/heartjnl-2017-311652

**Published:** 2017-08-05

**Authors:** Sean Lee Zheng, Fiona T Chan, Adam A Nabeebaccus, Ajay M Shah, Theresa McDonagh, Darlington O Okonko, Salma Ayis

**Affiliations:** 1 Cardiovascular Division, King’s College Hospital London, British Heart Foundation Centre of Research Excellence, London, UK; 2 Department of Cardiovascular Medicine, King’s College Hospital, London, UK; 3 Imperial College Healthcare NHS Trust, London, UK; 4 Department of Primary Care and Public Health Sciences, King’s College London, London, UK

**Keywords:** heart failure, preserved ejection fraction, mid-range ejection fraction, diastolic dysfunction, systematic review, meta-analysis

## Abstract

**Background:**

Clinical drug trials in patients with heart failure and preserved ejection fraction have failed to demonstrate improvements in mortality.

**Methods:**

We systematically searched Medline, Embase and the Cochrane Central Register of Controlled Trials for randomised controlled trials (RCT) assessing pharmacological treatments in patients with heart failure with left ventricular (LV) ejection fraction≥40% from January 1996 to May 2016. The primary efficacy outcome was all-cause mortality. Secondary outcomes were cardiovascular mortality, heart failure hospitalisation, exercise capacity (6-min walk distance, exercise duration, VO_2_ max), quality of life and biomarkers (B-type natriuretic peptide, N-terminal pro-B-type natriuretic peptide). Random-effects models were used to estimate pooled relative risks (RR) for the binary outcomes, and weighted mean differences for continuous outcomes, with 95% CI.

**Results:**

We included data from 25 RCTs comprising data for 18101 patients. All-cause mortality was reduced with beta-blocker therapy compared with placebo (RR: 0.78, 95%CI 0.65 to 0.94, p=0.008). There was no effect seen with ACE inhibitors, aldosterone receptor blockers, mineralocorticoid receptor antagonists and other drug classes, compared with placebo. Similar results were observed for cardiovascular mortality. No single drug class reduced heart failure hospitalisation compared with placebo.

**Conclusion:**

The efficacy of treatments in patients with heart failure and an LV ejection fraction≥40% differ depending on the type of therapy, with beta-blockers demonstrating reductions in all-cause and cardiovascular mortality. Further trials are warranted to confirm treatment effects of beta-blockers in this patient group.

## Introduction

Heart failure with preserved left ventricular (LV) ejection fraction (HFpEF) is a heterogeneous clinical syndrome defined by the presence of signs and symptoms of heart failure without evidence of reduced LV ejection fraction (typically considered as <40%).^1^ While significant advances have been made in the treatment of heart failure with reduced ejection fraction (HFrEF), randomised controlled trials (RCT) of pharmacological therapies in heart failure with an LV ejection fraction of 40% or more have been generally disappointing with no convincing demonstration of mortality or morbidity reduction. Updated guidelines recommend the use of diuretics for symptom relief and appropriate management of comorbidities (including hypertension), while acknowledging the absence of specific disease-modifying therapies in this condition.^1 2^

Although trial evidence demonstrating improvements in mortality have been inconsistent and largely neutral, several trials have suggested that drug therapy may improve exercise tolerance and quality of life.^3^ Since patients with HFpEF tend to be older with more comorbidities than their HFrEF counterparts,^4 5^ the efficacy of drug treatments might best be evaluated by their effects on hospitalisation, functional status, symptoms and quality of life.^1^

In this study, we aimed to systematically review the clinical trials of patients with HFpEF (defined as LV ejection fraction ≥40%), and identify treatment effects on mortality, heart failure hospitalisation, functional status and biomarker levels.

## Methods

This article has been reported in accordance with the Preferred Reporting Items for Systematic Reviews and Meta-Analyses.^6^ No published study protocol exists for this meta-analysis.

### Definition of heart failure with preserved ejection fraction

The latest European Society of Cardiology guidelines introduced the term heart failure with mid-range ejection fraction (HFmrEF), categorising an intermediate group of patients with an LV ejection fraction of between 40% and 49%, with HFpEF defined as an LV ejection fraction ≥50% with the same echocardiographic criteria.^1^ The American College of Cardiology defines HFpEF as an LV ejection fraction >40%, with anything from 41% to 49% as borderline HFpEF.^2^ While the terminology has changed in the process of this meta-analysis being undertaken, the aim of this study was to identify treatment effects in the group of patients with heart failure with LV ejection fraction ≥40%, for which no guideline-recommended therapies currently exist. In the HFpEF population, RCTs have used various LV ejection fraction cut-offs, ranging from 40% to 50%, and therefore data summarised in this meta-analysis will include patients in the mid-range and borderline group. Heart failure with LV ejection fraction ≥40% will henceforth be referred to as HFpEF.

### Search strategy and selection criteria

A systematic search of Medline, Embase and the Cochrane Central Register of Controlled Trials was performed using the search strategy documented in the online supplementary materials. Results were filtered for randomised controlled trials using predesigned and validated filters. The search was run on 1 May 2016, with results included from database inception to 1 May 2016. The search was rerun on 1 April 2017 and no additional articles were identified. The reference lists of included studies were searched for additional analyses. A systematic approach was used to identify systematic reviews and meta-analyses published during this period, which were hand-screened for additional trials.

10.1136/heartjnl-2017-311652.supp1 Supplementary file 1

Trials were considered eligible if they were (a) RCT; (b) enrolled participants with heart failure and documented LV ejection fraction ≥40%; (c) compared drug therapy with placebo, no treatment, diuretic treatment or standard medical treatment, with a minimum follow-up of at least 12 weeks and (d) provided information on prespecified primary and secondary end points that included all-cause mortality, cardiovascular mortality, heart failure hospitalisation, exercise capacity (6 min walk distance (6MWD), exercise duration, VO_2_ max), quality of life as measured using the Minnesota Living With Heart Failure Questionnaire (MLHFQ) and biomarkers (B-type natriuretic peptide (BNP), N-terminal pro-B-type natriuretic peptide (NT-proBNP)). Non-English language publications were excluded. We allowed secondary publications of included trials if they reported additional outcomes that were not present in the original article.

**Figure 1:**
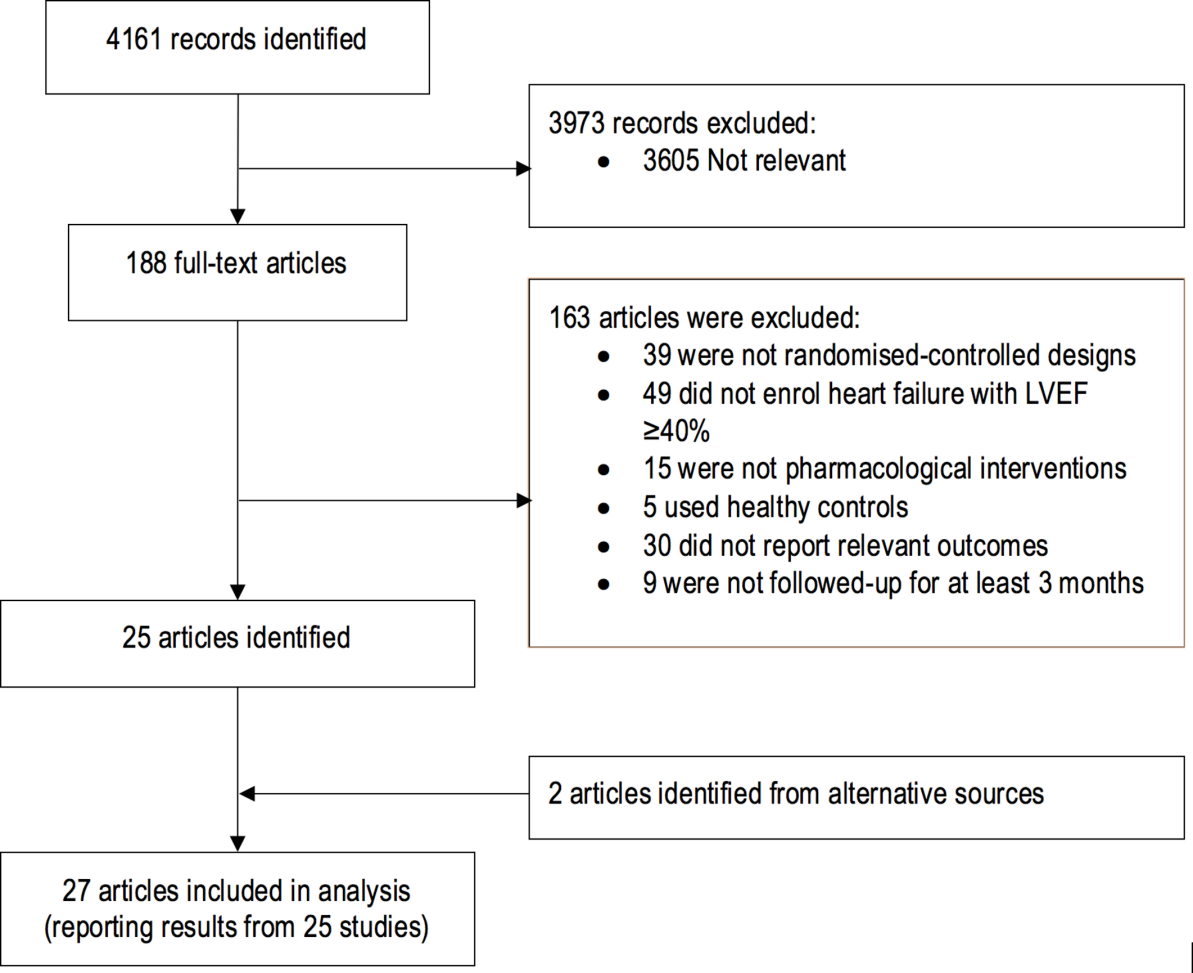
Study flow diagram of the trial selection process. LVEF, left ventricular ejection fraction.

After removal of duplicates, the title and abstracts of initial search results were screened for relevance. The full texts of remaining results were independently assessed by two authors (SLZ, FTC) for inclusion based on predetermined inclusion and exclusion criteria. The final list of included studies was decided by discussion between authors and required full agreement. No disagreements required resolution by a third reviewer. Study selection flow diagram is shown in figure 1.

### Data extraction and quality assessment

Data were extracted using piloted forms, independently and in duplicate by two authors (SLZ, FTC), and were transcribed onto a dedicated database. The data extracted from each report included baseline patient characteristics (age, sex, comorbidities, mean ejection fraction), study inclusion criteria, study drug and control treatment, follow-up duration and end point data.

The Cochrane Collaboration risk of bias tool was used to assess risk of bias. Disagreements in abstracted data were adjudicated by a third reviewer (AAN). Egger test was used to identify asymmetry of funnel plots for publication bias.

### Statistical analysis

Random-effects models were used to estimate pooled effect size from aggregate data.^7^ For binary outcomes, estimates were summarised as relative risk (RR) and 95% CI. For continuous outcomes, weighted mean difference (MD) and 95% CIs were calculated. Studies with no outcome events in the treatment or placebo groups, and therefore did not contribute to the risk ratios, were excluded from the RR pooled estimates, and were summarised using risk differences. Estimates and 95% CIs were graphically presented using Forest plots.^8^ χ^2^ test was used to compare differences between stratified subgroup RR.

Results were stratified by drug type (beta-blocker, ACE inhibitor, angiotensin receptor blocker (ARB), mineralocorticoid receptor antagonist (MRA), combined renin-angiotensin-aldosterone system (RAAS) antagonists and other drug types), duration of follow-up (3–12 months and >12 months), LV ejection fraction entry threshold (40%–49% and ≥50%) and mean LV ejection fraction (<60% and ≥60%). The original protocol did not specify stratifying by patient’s LV ejection fraction (40%–49% and ≥50%). No trials enrolled patients exclusively with LV ejection fraction between 40% and 49%, and no trials reported subgroup analyses of this group.

Between-study heterogeneity was assessed using I² statistics, which describes the percentage of variation across studies that is due to heterogeneity rather than chance.^9^ Publication bias was assessed if the number of trials was reasonable (10 or more) using funnel plots. No assessment was made for smaller number of trials due to the established poor performance of the test.^10^ Statistical analysis was performed using the software STATA V.14 and Review Manager V.5.3.

## Results

The electronic search identified 4161 articles that were screened and evaluated for eligibility based on title and abstract only (figure 1). After removal of duplicate (368) and non-relevant (3605) articles, 188 articles were evaluated in full-text for eligibility. A further 163 articles were excluded, and two additional eligible articles were identified from searching systematic reviews and reference lists. In all, 27 articles were included for meta-analysis, comprising data from 25 trials, with 28 separate comparisons (see online supplementary file 1). In total, 18 101 patients were randomised to either drug intervention or placebo, control or standard medical therapy. There were six beta-blocker trials enrolling 1299 patients, 5 ACE inhibitor (1305 participants), 6 ARB (9704 participants) and 5 MRA (4003 participants) trials. Other drugs tested include one digoxin (988 participants), two calcium channel blocker (242 participants), one sildenafil (216 participants), one sitaxsentan (192 participants) and one doxazosin (145 participants) trial. Seven studies used an LV ejection fraction threshold of 40%, and nine used LV ejection fraction thresholds of 45% and 50%. The inclusion criteria for Study of Effects of Nebivolol Intervention on Outcomes and Rehospitalization in Seniors With Heart Failure (SENIORS) included either an LV ejection fraction threshold of 35% or previous hospitalisation for heart failure regardless of ejection fraction, with subgroup analysis of patients with LV ejection fraction ≥40%, and so the study was included in the pooled analysis.^11^

### Effect of therapy on all-cause mortality, cardiovascular mortality and heart failure hospitalisation

#### Stratification by drug class

**Figure 2:**
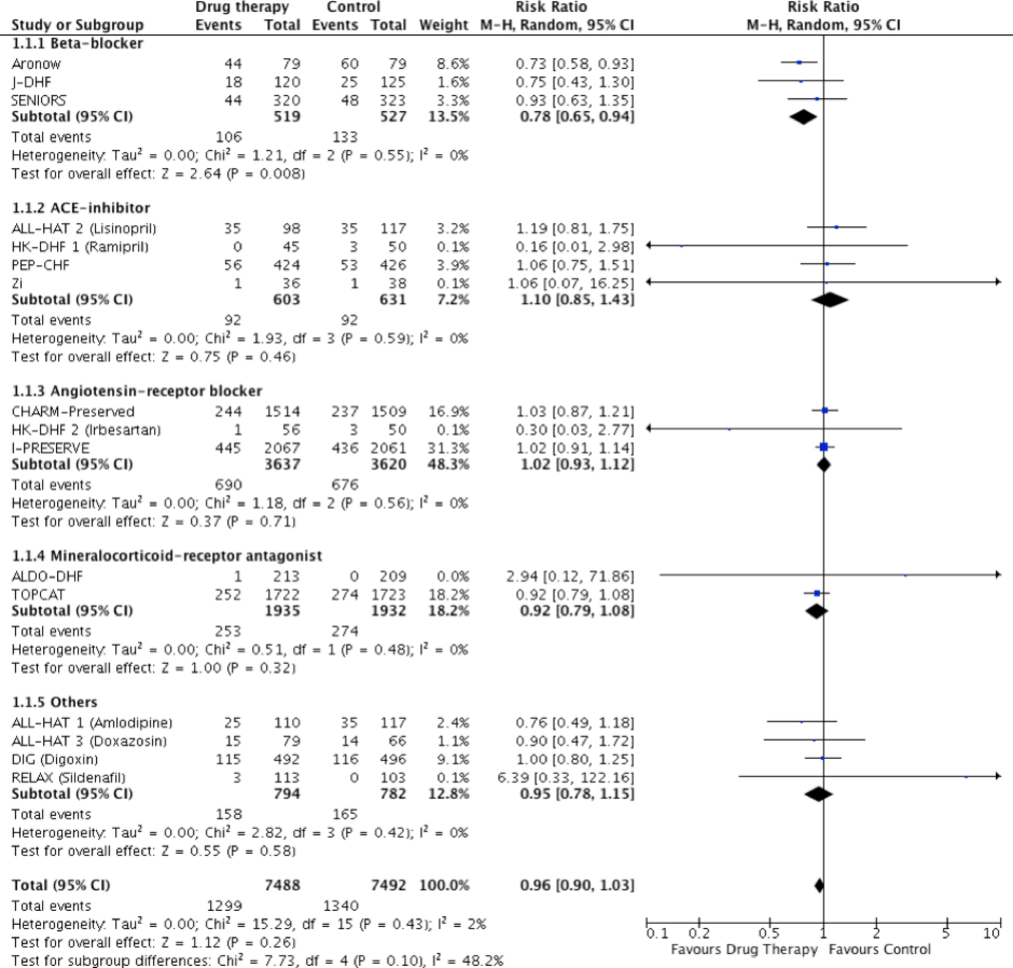
Pooled and individual estimates of relative risk (RR) and 95% CI of the primary outcome all-cause mortality for different therapies. Data are shown stratified by individual drug classes (beta-blockers, ACE inhibitors, angiotensin receptor blockers, mineralocorticoid receptor antagonists and other drug classes). Random-effects model used.

The search identified 16 studies that reported all-cause mortality events in one or both arms, which were pooled for analysis. Beta-blockers were the only pharmacological agent that reduced the risk of all-cause mortality compared with control (RR: 0.78, 95% CI: 0.65 to 0.94, p=0.008, n=1046)^11–13^ (figure 2, table 1). Pooled analysis of ACE inhibitor (RR: 1.10, 95% CI: 0.85 to 1.43, p=0.46, n=1234),^14–17^ ARB (RR: 1.02, 95% CI: 0.93 to 1.12, p=0.71, n=7257)^14 18–20^ and MRA (RR: 0.92, 95% CI: 0.79 to 1.08, p=0.32, n=3867)^21 22^ trials showed no effect on all-cause mortality compared with control. Pooled trials of drugs blocking the renin-angiotensin-aldosterone system (RAAS) (ACE inhibitor, ARB and MRA) did not reduce all-cause mortality (RR: 1.00, 95% CI 0.93 to 1.08, p=0.97, n=12 358) (see online supplementary figure 1A). Pooled effects of other drug types showed no difference (RR: 0.95, 95% CI: 078 to 1.15, p=0.58, n=1576).^16 23 24^

**Figure 3:**
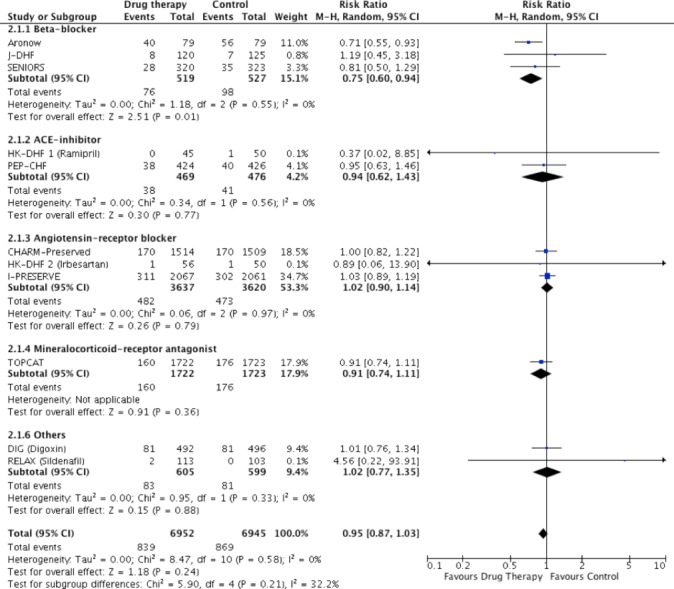
Pooled and individual estimates of relative risk (RR) and 95% CIs of the secondary outcome cardiovascular mortality for different therapies. Data are shown stratified by individual class blockers (beta-blockers, ACE inhibitors, angiotensin receptor blockers, mineralocorticoid receptor antagonists and other drug classes). Random-effects model used.

Cardiovascular mortality was reduced in beta-blocker therapy compared with controls (RR: 0.75, 95% CI: 0.60 to 0.94, p=0.01, n=1046), whereas ACE inhibitors (RR: 0.94, 95% CI: 0.62 to 1.43, p=0.77, n=945) and ARB (RR: 1.02, 95% CI: 0.90 to 1.14, p=0.79, n=7257) had no effect on cardiovascular mortality (figure 3). There was no effect of pooled RAAS blockade (RR 0.99, 95% CI 0.89 to 1.09, p=0.77) on cardiovascular mortality compared with controls (see online supplementary figure 2A).

**Figure 4:**
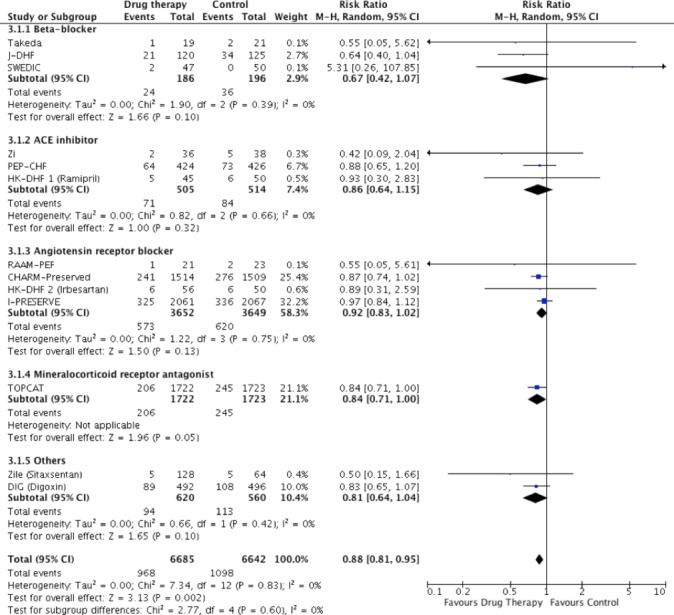
Pooled and individual estimates of relative risk (RR) and 95% CI of the secondary outcome heart failure hospitalisation for different therapies. Data are shown stratified by individual class blockers (beta-blockers, ACE inhibitors, angiotensin receptor blockers, mineralocorticoid receptor antagonists and other drug classes). Random-effects model used.

Pooled RAAS blockade reduced the risk for heart failure hospitalisation (RR=0.90, 95% CI: 0.82 to 0.98, p=0.01, n=11 765), though ACE inhibitors (RR: 0.86, 95% CI: 0.64 to 1.15, p=0.32, n=1019) and ARB (RR 0.92, 95% CI: 0.83 to 1.02, p=0.13, n=7301) had no effect individually (online supplementary figure 3A and figure 4). There was no effect of beta-blocker therapy on heart failure hospitalisation (RR: 0.67, 95% CI: 0.42 to 1.07, p=0.10, n=382) (figure 4). Only one trial of MRA (Treatment of Preserved Cardiac Function Heart Failure With an Aldosterone Antagonist trial (TOPCAT)^21^ reported cardiovascular mortality and heart failure hospitalisation outcomes (HR: 0.90, p=0.35 and HR: 0.83, p=0.04, respectively).

Meta-analysis of all trials revealed that pharmacotherapy did not improve all-cause (RR: 0.96, 95% CI: 0.90 to 1.03, p=0.26) or cardiovascular (RR: 0.95, 95% CI: 0.87 to 1.03, p=0.23) mortality, but reduced heart failure hospitalisation (RR: 0.88, 95% CI: 0.81 to 0.95, p=0.002). When all trials (including trials with no events in both arms) were considered using the mean weighted absolute risk difference, no significant difference was observed between treatment and control groups for all-cause mortality (Risk Difference (RD): −0.00%, 95% CI: −0.01% to 0.01%, p=0.99, n=15 340), or cardiovascular mortality (RD: −0.00%, 95% CI: −0.01% to 0.01%, p=0.70, n=14 257) (see online supplementary figure 1B and 2B). Heart failure hospitalisation showed a small but significant difference in risk favouring treatment (RD: −0.02%, 95% CI: −0.03 to −0.00%, p=0.005, n=13 491), driven by the treatment effect of RAAS blockade (see online supplementary figure 3B). Summary of effects for all primary and secondary outcomes by stratification is shown in online supplementary table 2.

#### Stratification by LV ejection fraction inclusion threshold

When analyses were stratified by LV ejection fraction inclusion threshold (40%–49%, ≥50%), neither group demonstrated significant differences in all-cause mortality, with no difference between subgroups (χ^2^=0.05, p=0.83) (see online supplementary figure 1C). Hospitalisation for heart failure was reduced in studies that used LV ejection fraction inclusion cut-offs of 40%–49% (RR: 0.88, 95% CI: 0.82 to 0.96, p=0.002), but not in studies using cut-offs of 50% or more (RR: 0.51, 95% CI: 0.18 to 1.48, p=0.22) (χ^2^=1.01, p=0.31). Only one study that reported cardiovascular mortality used an LV ejection fraction inclusion cut-off of 50% making a similar comparison not possible for this outcome.

#### Stratification by mean LV ejection fraction

Comparison of trials with a mean LV ejection fraction <60% (five trials, n=7688) with ≥60% (seven trials, n=6062) demonstrated that neither subgroup reached statistical significance for all-cause or cardiovascular mortality, with no difference between the subgroups (χ^2^=1.05, p=0.31) (online supplementary figures 1E and 2E, table 1). Heart failure hospitalisation was reduced in studies with a mean LV ejection fraction <60% (RR: 0.85, 95% CI 0.76 to 0.94, p=0.002), but not in studies with higher mean LV ejection fractions (RR: 0.92, 95% CI: 0.82 to 1.04, p=0.19) (χ^2^=1.03, p=0.31).

#### Stratification by follow-up duration

Trials with shorter follow-up (3–12 months) demonstrated statistically significant reductions in relative risk for both mortality outcomes (all-cause mortality: RR: 0.79, 95% CI: 0.66 to 0.95; p=0.01, cardiovascular mortality: RR: 0.71, 95% CI: 0.55 to 0.90, p=0.005) compared with those with longer follow-up (>12 months) (all-cause mortality: RR: 0.99, 95% CI: 0.92 to 1.06, p=0.77; cardiovascular mortality: RR: 0.99, 95% CI: 0.90 to 1.08, p=0.76) (see online supplementary figures 1D and 2D, table 1). Pharmacotherapy reduced heart failure hospitalisations in both shorter and longer trials, with a numerically greater reduction in risk in shorter trials: RR: 0.67, 95% CI: 0.48 to 0.94, p=0.02 and RR: 0.90, 95% CI: 0.82 to 0.98, p=0.02, respectively (χ^2^=2.70, p=0.10) (online supplementary figure 3D).

### Effect of therapy on exercise capacity

Ten studies (n=1870 patients) reported on 6MWD, eight studies (n=938) reported on exercise time after treatment and six studies (n=924) reported on VO_2_ max. There was no significant difference between groups for exercise time, VO_2_ max and 6MWD (see online supplementary figure 4).

### Effect of therapy on quality of life

Nine trials reported the treatment effects on quality of life as measured by the MLHFQ, including a total of 3510 patients (beta-blocker: 116 patients, ACE inhibitor: 166, ARB: 2460, MRA: 444, other: 324). Overall estimate showed that treatment resulted in better quality of life scores (MD: −1.63, 95% CI: −2.94 to −0.31, p=0.001) (see online supplementary figure 5).

### Effect of therapy on heart failure biomarkers

Thirteen trials reported BNP and NT-proBNP levels after treatment (see online supplementary table 3). Studies had a high degree of heterogeneity (I^2^=84.4%) and were not further analysed.

### Study quality and publication bias

Five studies were identified as high risk of bias using the Cochrane risk of bias tool, and the remainder low risk (see online supplementary materials **aterials**). The funnel plot for all-cause mortality, cardiovascular mortality and heart failure hospitalisation were symmetrical, providing no evidence of publication bias or small study effects (see online supplementary figure 6). Egger test did not identify asymmetry for any of the funnel plots.^25^

## Discussion

The results of this meta-analysis show significant reductions in all-cause and cardiovascular mortality in RCTs using beta-blockers, while RAAS blockade (using ACE inhibitor, ARB and MRA individually) demonstrated no effect on mortality. Improvements in functional outcomes and quality of life were not significantly or consistently demonstrated using pooled results. Heterogeneity within trials that reported biomarker outcomes was too high to allow comparison.

The effect of beta-blockade on mortality suggests favourable outcomes in patients with LV ejection fraction >40%. Similar benefits have been demonstrated in pooled analysis of observational studies and previous meta-analyses.^26–29^ Of the three beta-blocker trials, only one trial individually showed a significant reduction in mortality,^12^ and contributed the greatest weight to overall effects due to the high event rate. The two larger RCTs showed neutral results, although were both underpowered to detect effects on mortality.^11 13^ Notably, all three beta-blocker trials used an LV ejection fraction threshold of 40%, whereas trials using ACE inhibitor, ARB and MRA tended to use higher ejection fraction thresholds. The demonstrated reduction in mortality with beta-blockers may have been augmented by their effects on the HFmrEF population within these trials; a group that an emerging body of evidence suggests is more closely aligned with HFrEF.^30 31^ The beneficial effect of beta-blockers on mortality appears to be through preventing cardiovascular death, supported by a 25% reduction in cardiovascular mortality. We did not further investigate causes of cardiovascular death, although the pleiotropic effects of beta-blocker (such as its anti-arrhythmic properties) are likely to be important. Future sufficiently powered clinical trials and observational or registry data will be important in discerning if there is a true effect in patients with heart failure with an LV ejection fraction of 40% or more, and whether this benefit extends to those with LV ejection fraction >50%. While we did not demonstrate a benefit for beta-blockers in hospitalisation for heart failure, ‘hospitalisation’ should not be interpreted in isolation since mortality events will impact future hospitalisation events.

Given that patients with HFpEF tend to be older than their HFrEF counterparts, and are limited by disabling symptoms with poor quality of life, focus of therapy has shifted towards their effects on exercise tolerance and quality of life. This meta-analysis has shown that drug treatment is associated with a trend towards improvement in exercise time and significant although small improvement in quality of life measured using MLHFQ. Previous meta-analyses have been inconsistent in determining whether treatment improves exercise capacity,^3 32^ and the overall effects are unlikely to be clinically significant.

Interestingly, we showed that trials with shorter follow-up (between 3 and 12 months) demonstrated greater reductions in all clinical end points compared with longer trials. The importance of follow-up duration in HFpEF clinical trials is best highlighted by the Perindopril in Elderly People with Chronic Heart Failure (PEP-CHF) study. There was no significant difference in its primary outcome of combined all-cause mortality and heart failure hospitalisation (p=0.92) after 2.1 years of follow-up; however, a restrictive analysis at 1-year follow-up demonstrated an important effect in favour of perindopril (p=0.055). It is possible that as follow-up duration increases, cross-over of patients to unblinded study therapy (eg, for treatment of comorbidities) dilutes treatment effects and reduces study power to detect statistically meaningful differences. In this cohort of older and more comorbid patients, treatment efficacy could wane with time as patients develop mortality that is unaffected by treatments.

For hard clinical end points, our study did not find any difference when trials were stratified by LV ejection fraction thresholds (including patients with HFmrEF or not) nor by mean trial LV ejection fractions. Stratifying trials in this way results in smaller heterogeneous subgroups, and therefore evidence is not conclusive. The recently proposed HFmrEF category^1^ currently has no evidence-based treatments, and further clinical trials are required to identify whether this group will benefit from existing treatments used in HFrEF. Since it will be unlikely that dedicated RCTs will be undertaken in this group, meta-analysis using individual patient data and post hoc analysis of large trials will be important in shedding light on this less studied group. Indeed, post hoc analysis of Candesartan in Heart failure: Assessment of Reduction in Mortality and morbidity study (CHARM-Preserved) demonstrates a significant 24% reduction in CV death and time to first heart failure hospitalisation in patients with HFmrEF (HR: 0.76, 95% upper CI (UCI) 0.96, p=0.02).^33 34^ In TOPCAT, the potential efficacy of spironolactone was greatest in patients with LV ejection fractions <50% (HR: 0.72, UCI 1.05) compared with ≥60% (HR 0.97, UCI 1.23).^35^

HFpEF is a highly heterogeneous condition, with interactions between comorbidities likely to account for significant cardiovascular and non-cardiovascular mortality and morbidity.^4 5^ The effect of treatments on specific HFpEF phenotypes may identify subgroups that benefit. Latent class analysis of the I-PRESERVE data set identified a group of metabolic phenotype patients with HFpEF (high prevalence of diabetes, hyperlipidaemia and obesity) that benefited from irbesartan therapy.^36^ Indeed, further understanding of the pathophysiology of HFpEF will help to guide research and development of novel therapies.^1 5 37^

### Limitations

The aim of the meta-analysis is to determine treatment effects in patients with an LV ejection fraction ≥40%. At the time of undertaking the meta-analysis, these patients were defined as HFpEF, and so the nomenclature has been maintained in this article. Subsequently, there has been a change in definition with introduction of the HFmrEF category. As a result, pooled analysis is for patients with an LV ejection fraction ≥40%, and not ≥50%. None of the trials reported subgroup data on patients with an LV ejection fraction between 40% and 49%, and therefore we are unable to report on the treatment effect in these patients. Repeating the meta-analysis with individual patient data will provide greater insights into this intermediate group.

Analyses were stratified by drug class (beta-blockers, RAAS antagonists and other classes). The drugs in the other category include a range of vasodilators, calcium channel blockers and digoxin and do not exert their effects through the same mechanisms. We have also made aggregate analyses based on all pharmacological agents, which has limitations, although was used in previous HFpEF meta-analyses due to the small number of trials in each individual drug class.^3^ Where data are available, we have sought to draw conclusions from comparison within drug classes to allow meaningful clinical conclusions to be drawn.

The general limitations associated with meta-analyses extends to this current work. We are limited by availability and reporting of data.^38^ Risk ratios were used as only a small number of trials reported HRs, which introduces bias associated with comparing outcomes in trials of different lengths.

### Conclusions

In trials enrolling patients with HFpEF, defined using an LV ejection fraction ≥40%, beta-blockers reduce all-cause and cardiovascular mortality by 22% and 25%, respectively. There was no significant effect of ACE inhibitors, ARB or MRA on the same outcomes. The effect of treatments on functional and quality of life outcomes was limited. Further adequately powered RCTs in beta-blocker therapy in this patient group is warranted to confirm this finding.

**Key messages**

**What is already known on this subject?**

There have been no individual large randomised controlled trials which demonstrate improvements in clinical outcomes for patients with heart failure and preserved ejection fraction. This is represented in the latest European Society of Cardiology Heart Failure guidelines, which advocate treatment of comorbidities and symptoms.

**What might this study add?**

This meta-analysis shows that pooled analysis of beta-blocker trials demonstrates reductions in all-cause and cardiovascular mortality by 22% and 25%, respectively, with no pooled effect seen in ACE inhibitor, angiotensin receptor blocker and mineralocorticoid antagonist trials. There may also be a small benefit in terms of quality of life with several of the drug classes tested, although no difference in functional outcomes.

**How might this impact on clinical practice?**

Clinicians should consider using beta-blockers when there is an existing indication for them. There may be benefit in repeating a randomised controlled trial of beta-blockers in heart failure with preserved left ventricular ejection fraction with contemporary patient cohorts.

**Table 1:** Summary of effects for all-cause mortality, cardiovascular mortality and heart failure hospitalisation

Outcome	All trials	Drug classes			Follow-up duration		Entry LV ejection fraction threshold		Mean LV ejection fraction	
		Beta-blockers	RAAS antagonists	Other	3–12 months	>12 months	40%–49%	≥50%	<60%	≥60%
All-cause mortality	0.96 (0.90 to 1.03)	0.78 (0.65 to 0.94) p=0.008	1.00 (0.93 to 1.08)	0.95 (0.78 to 1.15)	0.79 (0.66 to 0.95) p=0.01	0.99 (0.92 to 1.06)	0.96 (0.88 to 1.03)	0.99 (0.74 to 1.32)	0.93 (0.82 to 1.05)	1.01 (0.90 to 1.12)
Cardiovascular mortality	0.95 (0.87 to 1.03)	0.75 (0.60 to 0.94) p=0.01	0.99 (0.89 to 1.09)	1.01 (0.76 to 1.34)	0.71 (0.55 to 0.90) p=0.005	0.99 (0.90 to 1.08)	0.95 (0.87 to 1.03)	-	0.90 (0.78 to 1.05)	1.02 (0.89 to 1.17)
Heart failure hospitalisation	0.88 (0.81 to 0.95) p=0.002	0.67 (0.42 to 1.07)	0.90 (0.82 to 0.98) p=0.01	0.81 (0.64 to 1.04)	0.67 (0.48 to 0.94) p=0.02	0.90 (0.82 to 0.98) p=0.02	0.88 (0.82 to 0.96) p=0.002	0.51 (0.18 to 1.48)	0.85 (0.76 to 0.94) p=0.002	0.92 (0.82 to 1.04)

Data presented as risk ratios (for all-cause and cardiovascular mortality and hospitalisation outcomes) or mean difference (exercise capacity, 6MWD, VO_2_ max and MLHFQ), with 95% CI and I^2^ statistic. p values included for analyses that reached statistical significance at p=0.05. RAAS blockers include all trials using ACE inhibitors, angiotensin receptor blockers and mineralocorticoid (each class individually had no effect on all-cause mortality, cardiovascular mortality or heart failure hospitalisation). Only one trial that reported cardiovascular mortality had an entry LV ejection fraction ≥50%. Only one trial with LV ejection fraction threshold ≥50% reported cardiovascular mortality.

6MWD, 6 min walk distance; LV, left ventricular; MLHFQ, Minnesota Living With Heart Failure Questionnaire; RAAS, renin-angiotensin-aldosterone system.
