# Supplementary material for: Drug treatment effects on outcomes in heart failure with preserved ejection fraction: a systematic review and meta-analysis
Source: Heart. 2017 Aug 5;104(5):407–15. doi: 10.1136/heartjnl-2017-311652 (PMC5861385; doi:10.1136/heartjnl-2017-311652)
Supplement: Supplementary file 1 [file heartjnl-2017-311652supp001.pdf]

Supplementary materials

Search strategy Page 2

Cochrane Risk of Bias Assessment Page 4

Figure 1 - All-cause mortality Page 5

A. Stratified by class (pooled RAAS antagonists) (RR)
B. Stratified by drug class (RD)
C. Stratified by entry LV ejection fraction threshold (40 to 49%, ≥50%)
D. Stratified by follow-up duration (3-12months vs. >12months)
E. Stratified by mean LV ejection fraction (<60% and ≥60%)

Figure 2 - Cardiovascular mortality Page 10

A. By drug type (beta-blockers, RAAS antagonists and other) (RR)
B. By drug type (beta-blockers, RAAS antagonists and other) (RD)
C. By entry LVEF cut-off (>40 and 45%, >50%)
D. By follow-up duration (3-12months vs. >12months)
E. Stratified by mean LV ejection fraction (<60% and ≥60%)

Figure 3 - Heart failure hospitalisation – mean weighted difference Page 15

A. By drug type (beta-blockers, RAAS antagonists and other) (RR)
B. By drug type (beta-blockers, RAAS antagonists and other) (RD)
C. By entry LVEF cut-off (>40 and 45%, >50%)
D. By follow-up duration (3-12months vs. >12months)
E. Stratified by mean LV ejection fraction (<60% and ≥60%)

Figure 4 – Exercise outcomes Page 20

Figure 5 – Quality of Life Page 22

Figure 6 – Funnel plot Page 23

A. All-cause mortality
B. Cardiovascular mortality
C. Heart failure hospitalisation

Table 1 - Study characteristics for randomised controlled trials Page 24

Table 2 - Summary of effects for all primary and secondary outcomes Page 27

Table 3 – Overview of changes to BNP and NT-pro BNP following treatment in included trials Page 28

Reference List Page 28

Search Strategy

Medline, Embase and CENTRAL were searched using the following keywords:

- *heart failure* AND *normal ejection fraction*
- *heart failure* AND *preserved cardiac function*
- *heart failure* AND *preserved ejection fraction*
- *diastolic heart failure*
- *diastolic dysfunction*
- *HFpEF*
- *HFnEF*

Cochrane search filter for RCTs was applied

Source: (http://work.cochrane.org/rct-filters-different-databases)

**Medline**	**EMBASE**	**Cochrane CENTRAL**
1. Heart failure<br>2. Normal ejection fraction<br>3. Preserved cardiac function<br>4. Preserved ejection fraction<br>5. 1 and 2<br>6. 1 and 3<br>7. 1 and 4<br>8. Diastolic heart failure<br>9. Diastolic dysfunction<br>10. HFPEF<br>11. HFNEF<br>12. Or/5-11<br>13. Randomized Controlled Trials as Topic/<br>14. Randomized controlled trial/<br>15. Random Allocation/<br>16. Double Blind Method/<br>17. Single Blind Method/<br>18. Clinical trial/<br>19. Clinical trial, phase i.pt<br>20. Clinical trial, phase ii.pt<br>21. Clinical trial, phase	1. Heart failure<br>2. Normal ejection fraction<br>3. Preserved cardiac function<br>4. Preserved ejection fraction<br>5. 1 and 2<br>6. 1 and 3<br>7. 1 and 4<br>8. Diastolic heart failure<br>9. Diastolic dysfunction<br>10. HFPEF<br>11. HFNEF<br>12. Or/5-11<br>13. Clinical trial/<br>14. Randomized controlled trial/<br>15. Randomization/<br>16. Single blind procedure/<br>17. Double blind procedure/<br>18. Crossover procedure/<br>19. Placebo/<br>20. Randomi?ed controlled trial$.tw.<br>21. Rct.tw.<br>22. Random allocation.tw.	In "Trials"<br>1. heart failure<br>2. preserved ejection fraction<br>3. preserved cardiac function<br>4. normal ejection fraction<br>5. 1 and 2<br>6. 1 and 3<br>7. 1 and 4<br>8. "diastolic heart failure"<br>9. "diastolic dysfunction"<br>10. "HFNEF"<br>11. "HFPEF"<br>12. 5 or 6 or 7 or 8 or 9 or 10 or 11

iii.pt
22. Clinical trial, phase iv.pt
23. Controlled clinical trial.pt
24. Randomized controlled trial.pt
25. Multicenter study.pt
26. Clinical trial.pt
27. Exp Clinical Trials as topic/
28. Or/13-27
29. (clinical adj trial$).tw
30. ((singl$ or doubl$ or treb$ or tripl$) adj (blind$3 or mask$3)).tw
31. Placebos/
32. Placebo$.tw
33. Randomly allocated.tw
34. (allocated adj2 random$).tw
35. Or/29-34
36. 28 or 35
37. Case report.tw
38. Letter/
39. Historical article/
40. Or/37-39
41. 36 not 40
42. 12 and 41

23. Randomly allocated.tw.
24. Allocated randomly.tw.
25. (allocated adj2 random).tw.
26. Single blind$.tw.
27. Double blind$.tw.
28. ((treble or triple) adj (blind$).tw.
29. Placebo$.tw.
30. Prospective study/
31. Or/13-30
32. Case study/
33. Case report.tw.
34. Abstract report/ or letter/
35. Or/32-34
36. 31 not 35
37. 12 and 36

Cochrane risk of bias assessment

Author (Trial)	Selection bias	Performance bias	Detection bias	Reporting bias	Attrition bias	Overall risk of bias
Ahmed (DIG)	Low	Low	Low	Low	Low	Low
Aronow	Unclear	High	Low	Low	Low	High
Bergstrom (SWEDIC)	Unclear	Low	Low	Low	Low	Low
Cleland (PEP-CHF)	Low	Low	Low	Low	Unclear	Low
Conraads	Low	Low	Low	Low	Low	Low
Davis (ALLHAT)	Unclear	Low	Low	Low	Low	Low
Deswal (RAAM-PEF)	Unclear	Low	Low	Low	Low	Low
Edelmann (ALDO-DHF)	Low	Low	Low	Low	Low	Low
Hung	Low	Low	Low	Low	Low	Low
Kasama	Low	Low	Low	Low	Low	Low
Kitzman	Low	Low	Low	Low	Low	Low
Kurrelmeyer	Low	Low	Low	Low	Low	Low
Little	Low	Low	Low	Low	Low	Low
Mak	Low	High	Low	Low	Low	High
Massie (I-PRESERVE)	Low	Low	Low	Low	Low	Low
Parthasarathy	Low	Low	Low	Low	Low	Low
Pitt (TOPCAT)	Low	Low	Low	Low	Low	Low
Rector (I-PRESERVE)	Low	Low	Low	Low	Low	Low
Redfield (RELAX)	Low	Low	Low	Low	Low	Low
Takeda	Unclear	High	Unclear	Low	Low	High
Van Veldhuisen (SENIORS)	Low	Low	Low	Low	Low	Low
Yamamoto (J-DHF)	Unclear	High	Low	Low	Low	High
Yip (HK-DHF)	Low	High	Low	Low	Unclear	High
Yusuf (CHARM-preserved)	Low	Low	Low	Low	Low	Low
Zi	Unclear	Low	Low	Low	Low	Low
Zile	Unclear	Low	Low	Low	Low	Low

Supplementary Figure 1A: Pooled and individual estimates of relative risk (RR) and 95% confidence intervals (CI) of the primary outcome all cause-mortality for different drug classes. Data is shown stratified by beta-blockers, renin-angiotensin-aldosterone antagonists (RAAS) [which includes Angiotensin Converting Enzyme (ACE) inhibitors, Angiotensin Receptor Blockers (ARB), and Mineralocorticoid Receptor Antagonists(MRA)], and other drug classes. Random effects model used.

Study or Subgroup	Drug therapy Events	Drug therapy Total	Control Events	Control Total	Weight	Risk Ratio M-H, Random, 95% CI
**1.2.1 Beta-blocker**						
Aronow	44	79	60	79	8.6%	0.73 [0.58, 0.93]
J-DHF	18	120	25	125	1.6%	0.75 [0.43, 1.30]
SENIORS	44	320	48	323	3.3%	0.93 [0.63, 1.35]
**Subtotal (95% CI)**		**519**		**527**	**13.5%**	**0.78 [0.65, 0.94]**
Total events	106		133			
Heterogeneity: $Tau^2 = 0.00$; $Chi^2 = 1.21$, df = 2 (P = 0.55); $I^2 = 0\%$						
Test for overall effect: Z = 2.64 (P = 0.008)						
**1.2.2 Renin-angiotensin-aldosterone system blockade**						
ALDO-DHF	1	213	0	209	0.0%	2.94 [0.12, 71.86]
ALL-HAT 2 (Lisinopril)	35	98	35	117	3.2%	1.19 [0.81, 1.75]
CHARM-Preserved	244	1514	237	1509	16.9%	1.03 [0.87, 1.21]
HK-DHF 1 (Ramipril)	0	45	3	50	0.1%	0.16 [0.01, 2.98]
HK-DHF 2 (Irbesartan)	1	56	3	50	0.1%	0.30 [0.03, 2.77]
I-PRESERVE	445	2067	436	2061	31.3%	1.02 [0.91, 1.14]
PEP-CHF	56	424	53	426	3.9%	1.06 [0.75, 1.51]
TOPCAT	252	1722	274	1723	18.2%	0.92 [0.79, 1.08]
Zi	1	36	1	38	0.1%	1.06 [0.07, 16.25]
**Subtotal (95% CI)**		**6175**		**6183**	**73.7%**	**1.00 [0.93, 1.08]**
Total events	1035		1042			
Heterogeneity: $Tau^2 = 0.00$; $Chi^2 = 5.28$, df = 8 (P = 0.73); $I^2 = 0\%$						
Test for overall effect: Z = 0.03 (P = 0.97)						
**1.2.3 Others**						
ALL-HAT 1 (Amlodipine)	25	110	35	117	2.4%	0.76 [0.49, 1.18]
ALL-HAT 3 (Doxazosin)	15	79	14	66	1.1%	0.90 [0.47, 1.72]
DIG (Digoxin)	115	492	116	496	9.1%	1.00 [0.80, 1.25]
RELAX (Sildenafil)	3	113	0	103	0.1%	6.39 [0.33, 122.16]
**Subtotal (95% CI)**		**794**		**782**	**12.8%**	**0.95 [0.78, 1.15]**
Total events	158		165			
Heterogeneity: $Tau^2 = 0.00$; $Chi^2 = 2.82$, df = 3 (P = 0.42); $I^2 = 0\%$						
Test for overall effect: Z = 0.55 (P = 0.58)						
**Total (95% CI)**		**7488**		**7492**	**100.0%**	**0.96 [0.90, 1.03]**
Total events	1299		1340			
Heterogeneity: $Tau^2 = 0.00$; $Chi^2 = 15.29$, df = 15 (P = 0.43); $I^2 = 2\%$						
Test for overall effect: Z = 1.12 (P = 0.26)						
Test for subgroup differences: $Chi^2 = 6.03$, df = 2 (P = 0.05), $I^2 = 66.9\%$						

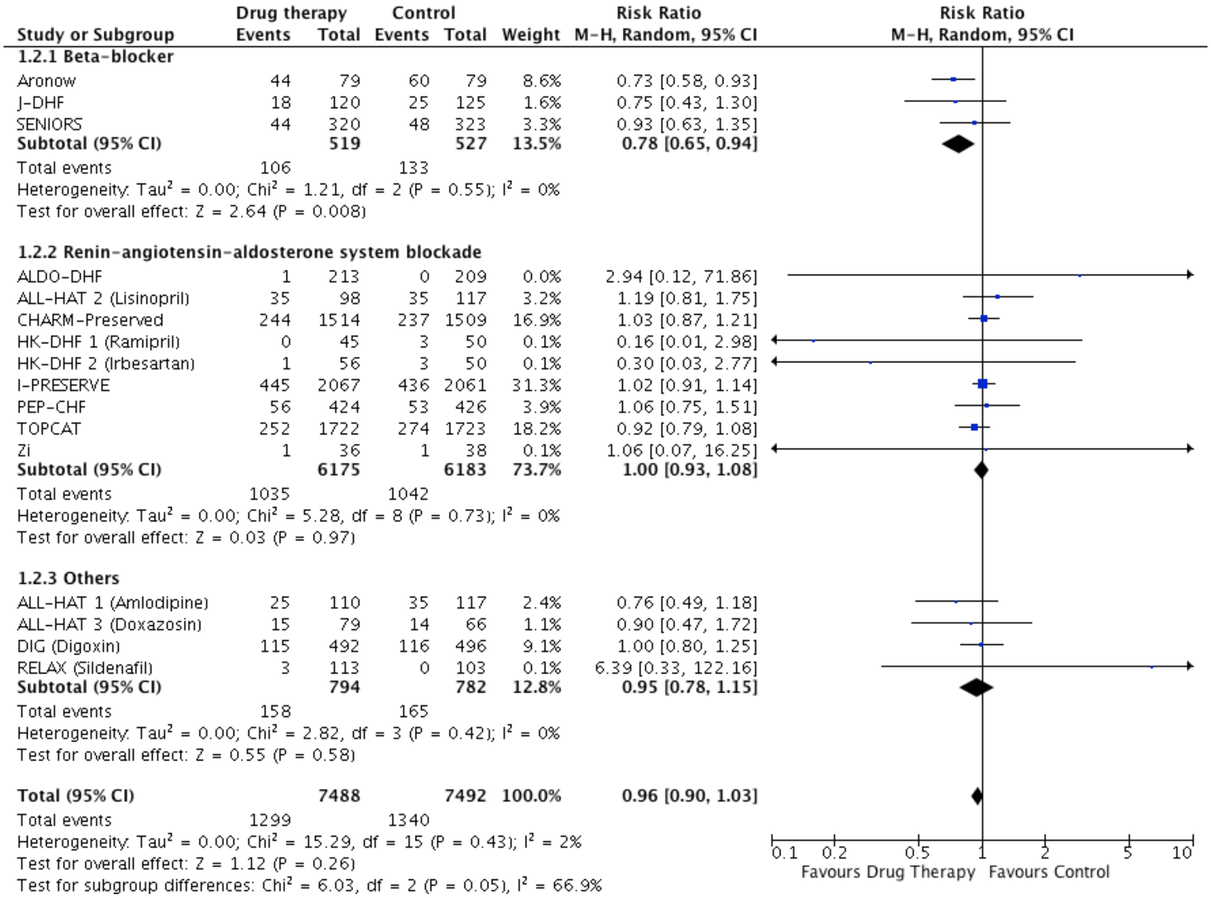

Supplementary Figure 1B: Pooled and individual estimates of risk difference (RD) and 95% CI of the primary outcome all cause-mortality for different drug classes. Data is shown stratified by beta-blockers, ACE inhibitors, ARB, and MRA, and other drug classes. Random effects model used.

Study or Subgroup	Drug therapy Events	Drug therapy Total	Control Events	Control Total	Weight	Risk Difference M-H, Random, 95% CI
**1.3.1 Beta-blocker**						
Aronow	44	79	60	79	0.3%	-0.20 [-0.35, -0.06]
ELANDD	0	57	0	59	6.2%	0.00 [-0.03, 0.03]
J-DHF	18	120	25	125	0.8%	-0.05 [-0.14, 0.04]
SENIORS	44	320	48	323	2.3%	-0.01 [-0.07, 0.04]
SWEDIC	0	47	0	50	4.4%	0.00 [-0.04, 0.04]
Takeda	0	19	0	21	0.8%	0.00 [-0.09, 0.09]
**Subtotal (95% CI)**		**642**		**657**	**14.8%**	**-0.02 [-0.07, 0.02]**
Total events	106		133			
Heterogeneity: Tau² = 0.00; Chi² = 16.05, df = 5 (P = 0.007); I² = 69%						
Test for overall effect: Z = 1.00 (P = 0.32)						
**1.3.2 ACE-inhibitor**						
ALL-HAT 2 (Lisinopril)	35	98	35	117	0.4%	0.06 [-0.07, 0.18]
HK-DHF 1 (Ramipril)	0	45	3	50	1.2%	-0.06 [-0.14, 0.02]
Kitzman	0	25	0	34	1.6%	0.00 [-0.07, 0.07]
PEP-CHF	56	424	53	426	3.4%	0.01 [-0.04, 0.05]
Zi	1	36	1	38	1.3%	0.00 [-0.07, 0.08]
**Subtotal (95% CI)**		**628**		**665**	**7.9%**	**-0.00 [-0.03, 0.03]**
Total events	92		92			
Heterogeneity: Tau² = 0.00; Chi² = 4.04, df = 4 (P = 0.40); I² = 1%						
Test for overall effect: Z = 0.16 (P = 0.87)						
**1.3.3 Angiotensin-receptor blocker**						
CHARM-Preserved	244	1514	237	1509	9.7%	0.00 [-0.02, 0.03]
HK-DHF 2 (Irbesartan)	1	56	3	50	1.2%	-0.04 [-0.12, 0.03]
I-PRESERVE	445	2067	436	2061	10.5%	0.00 [-0.02, 0.03]
**Subtotal (95% CI)**		**3637**		**3620**	**21.5%**	**0.00 [-0.02, 0.02]**
Total events	690		676			
Heterogeneity: Tau² = 0.00; Chi² = 1.43, df = 2 (P = 0.49); I² = 0%						
Test for overall effect: Z = 0.15 (P = 0.88)						
**1.3.4 Mineralocorticoid-receptor antagonist**						
ALDO-DHF	1	213	0	209	34.1%	0.00 [-0.01, 0.02]
Kurrelmeyer	0	24	0	24	1.1%	0.00 [-0.08, 0.08]
TOPCAT	252	1722	274	1723	11.4%	-0.01 [-0.04, 0.01]
**Subtotal (95% CI)**		**1959**		**1956**	**46.7%**	**-0.00 [-0.03, 0.02]**
Total events	253		274			
Heterogeneity: Tau² = 0.00; Chi² = 5.47, df = 2 (P = 0.06); I² = 63%						
Test for overall effect: Z = 0.19 (P = 0.85)						
**1.3.5 Others**						
ALL-HAT 1 (Amlodipine)	25	110	35	117	0.5%	-0.07 [-0.19, 0.04]
ALL-HAT 3 (Doxazosin)	15	79	14	66	0.4%	-0.02 [-0.15, 0.11]
DIG (Digoxin)	115	492	116	496	2.5%	-0.00 [-0.05, 0.05]
RELAX (Sildenafil)	3	113	0	103	5.7%	0.03 [-0.01, 0.06]
**Subtotal (95% CI)**		**794**		**782**	**9.1%**	**0.00 [-0.04, 0.05]**
Total events	158		165			
Heterogeneity: Tau² = 0.00; Chi² = 5.39, df = 3 (P = 0.15); I² = 44%						
Test for overall effect: Z = 0.03 (P = 0.98)						
**Total (95% CI)**		**7660**		**7680**	**100.0%**	**-0.00 [-0.01, 0.01]**
Total events	1299		1340			
Heterogeneity: Tau² = 0.00; Chi² = 20.46, df = 20 (P = 0.43); I² = 2%						
Test for overall effect: Z = 0.01 (P = 0.99)						
Test for subgroup differences: Chi² = 1.00, df = 4 (P = 0.91), I² = 0%						

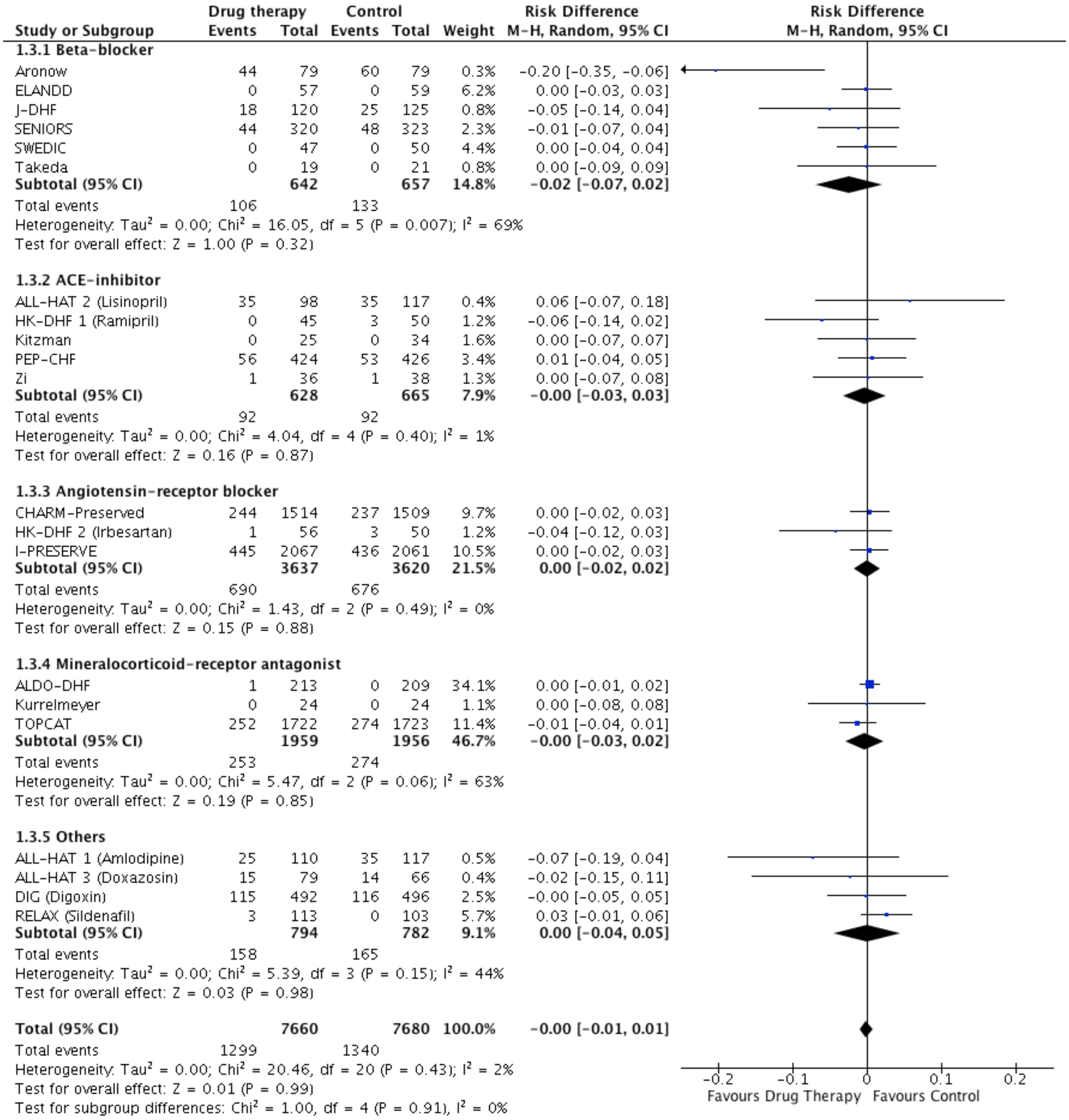

Supplementary Figure 1C: Pooled and individual estimates of relative risk (RR) and 95% CI of the primary outcome all cause-mortality stratified by entry LV ejection fraction threshold: 40 to 49% (trials which include patients with HFmrEF), and ≥50% (trials which include only HFpEF). Random effects model used.

Study or Subgroup	Drug therapy Events	Drug therapy Total	Control Events	Control Total	Weight	Risk Ratio M-H, Random, 95% CI
**1.4.1 Trial LV ejection fraction entry cut-off: 40 to 49%**						
Aronow	44	79	60	79	8.6%	0.73 [0.58, 0.93]
CHARM-Preserved	244	1514	237	1509	16.9%	1.03 [0.87, 1.21]
DIG (Digoxin)	115	492	116	496	9.1%	1.00 [0.80, 1.25]
HK-DHF 1 (Ramipril)	0	45	3	50	0.1%	0.16 [0.01, 2.98]
HK-DHF 2 (Irbesartan)	1	56	3	50	0.1%	0.30 [0.03, 2.77]
I-PRESERVE	445	2067	436	2061	31.3%	1.02 [0.91, 1.14]
J-DHF	18	120	25	125	1.6%	0.75 [0.43, 1.30]
PEP-CHF	56	424	53	426	3.9%	1.06 [0.75, 1.51]
SENIORS	44	320	48	323	3.3%	0.93 [0.63, 1.35]
TOPCAT	252	1722	274	1723	18.2%	0.92 [0.79, 1.08]
Zi	1	36	1	38	0.1%	1.06 [0.07, 16.25]
**Subtotal (95% CI)**		**6875**		**6880**	**93.1%**	**0.96 [0.88, 1.03]**
Total events	1220		1256			
Heterogeneity: Tau² = 0.00; Chi² = 10.88, df = 10 (P = 0.37); I² = 8%						
Test for overall effect: Z = 1.16 (P = 0.25)						
**1.4.2 Trial LV ejection fraction entry cut-off: ≥50%**						
ALDO-DHF	1	213	0	209	0.0%	2.94 [0.12, 71.86]
ALL-HAT 1 (Amlodipine)	25	110	35	117	2.4%	0.76 [0.49, 1.18]
ALL-HAT 2 (Lisinopril)	35	98	35	117	3.2%	1.19 [0.81, 1.75]
ALL-HAT 3 (Doxazosin)	15	79	14	66	1.1%	0.90 [0.47, 1.72]
RELAX (Sildenafil)	3	113	0	103	0.1%	6.39 [0.33, 122.16]
**Subtotal (95% CI)**		**613**		**612**	**6.9%**	**0.99 [0.74, 1.32]**
Total events	79		84			
Heterogeneity: Tau² = 0.01; Chi² = 4.37, df = 4 (P = 0.36); I² = 8%						
Test for overall effect: Z = 0.08 (P = 0.94)						
**Total (95% CI)**		**7488**		**7492**	**100.0%**	**0.96 [0.90, 1.03]**
Total events	1299		1340			
Heterogeneity: Tau² = 0.00; Chi² = 15.29, df = 15 (P = 0.43); I² = 2%						
Test for overall effect: Z = 1.12 (P = 0.26)						
Test for subgroup differences: Chi² = 0.05, df = 1 (P = 0.83), I² = 0%						

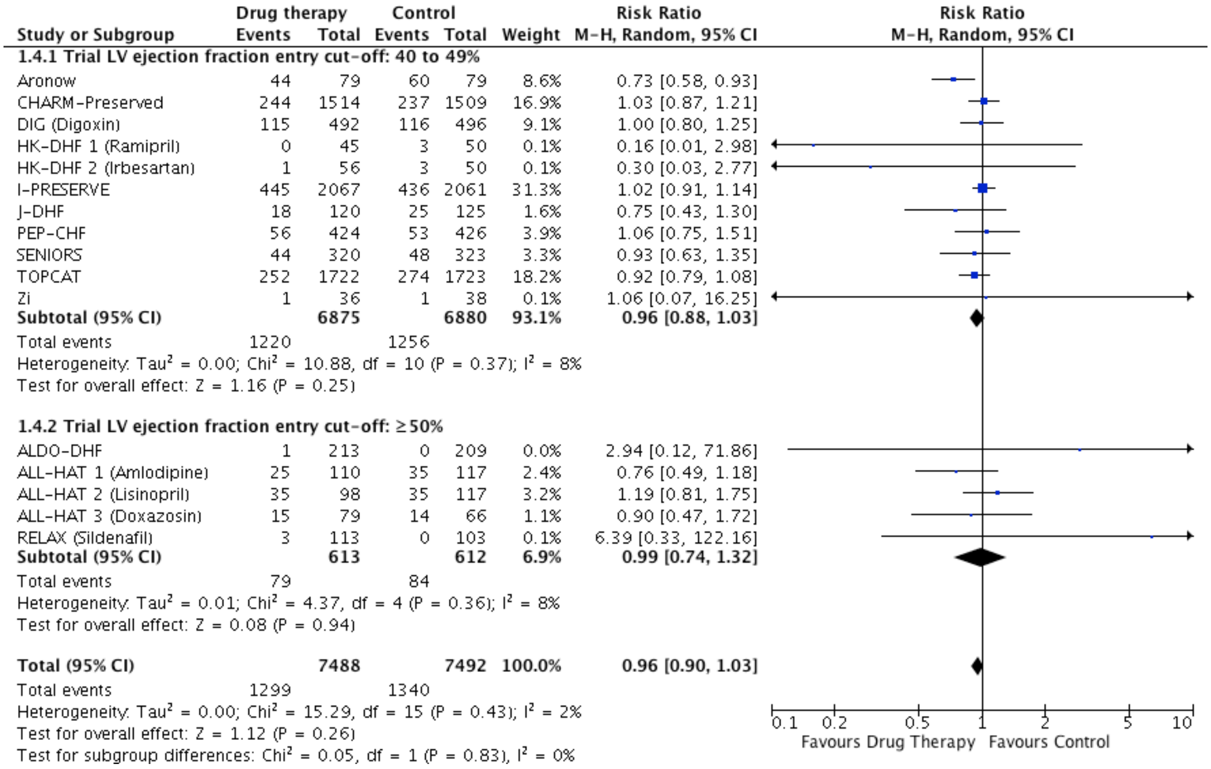

Supplementary Figure 1D: Pooled and individual estimates of relative risk (RR) and 95% CI of the primary outcome all cause-mortality stratified by follow-up duration: between 3 to 12 months and greater than 12 months. Random effects model used. PEP-CHF reported outcomes at 12 months follow-up in addition to end of follow-up (mean 2.1 years).

Study or Subgroup	Drug therapy Events	Drug therapy Total	Control Events	Control Total	Weight	Risk Ratio M-H, Random, 95% CI
**1.5.1 Follow-up: 3 to 12 months**						
ALDO-DHF	1	213	0	209	0.0%	2.94 [0.12, 71.86]
Aronow	44	79	60	79	8.2%	0.73 [0.58, 0.93]
HK-DHF 1 (Ramipril)	0	45	3	50	0.1%	0.16 [0.01, 2.98]
HK-DHF 2 (Irbesartan)	1	56	3	50	0.1%	0.30 [0.03, 2.77]
PEP-CHF (12 months)	17	424	19	426	1.1%	0.90 [0.47, 1.71]
RELAX (Sildenafil)	3	113	0	103	0.1%	6.39 [0.33, 122.16]
SENIORS	44	320	48	323	3.1%	0.93 [0.63, 1.35]
Zi	1	36	1	38	0.1%	1.06 [0.07, 16.25]
**Subtotal (95% CI)**		**1286**		**1278**	**12.7%**	**0.79 [0.66, 0.95]**
Total events	111		134			
Heterogeneity: $Tau^2 = 0.00$; $Chi^2 = 6.00$, df = 7 (P = 0.54); $I^2 = 0\%$						
Test for overall effect: Z = 2.45 (P = 0.01)						
**1.5.2 Follow-up: >12 months**						
ALL-HAT 1 (Amlodipine)	25	110	35	117	2.3%	0.76 [0.49, 1.18]
ALL-HAT 2 (Lisinopril)	35	98	35	117	3.0%	1.19 [0.81, 1.75]
ALL-HAT 3 (Doxazosin)	15	79	14	66	1.1%	0.90 [0.47, 1.72]
CHARM-Preserved	244	1514	237	1509	16.6%	1.03 [0.87, 1.21]
DIG (Digoxin)	115	492	116	496	8.7%	1.00 [0.80, 1.25]
I-PRESERVE	445	2067	436	2061	32.5%	1.02 [0.91, 1.14]
J-DHF	18	120	25	125	1.5%	0.75 [0.43, 1.30]
PEP-CHF (2.1 years)	56	424	53	424	3.6%	1.06 [0.74, 1.50]
TOPCAT	252	1722	274	1723	18.0%	0.92 [0.79, 1.08]
**Subtotal (95% CI)**		**6626**		**6638**	**87.3%**	**0.99 [0.92, 1.06]**
Total events	1205		1225			
Heterogeneity: $Tau^2 = 0.00$; $Chi^2 = 4.72$, df = 8 (P = 0.79); $I^2 = 0\%$						
Test for overall effect: Z = 0.29 (P = 0.77)						
**Total (95% CI)**		**7912**		**7916**	**100.0%**	**0.96 [0.90, 1.03]**
Total events	1316		1359			
Heterogeneity: $Tau^2 = 0.00$; $Chi^2 = 15.30$, df = 16 (P = 0.50); $I^2 = 0\%$						
Test for overall effect: Z = 1.15 (P = 0.25)						
Test for subgroup differences: $Chi^2 = 4.75$, df = 1 (P = 0.03), $I^2 = 79.0\%$						

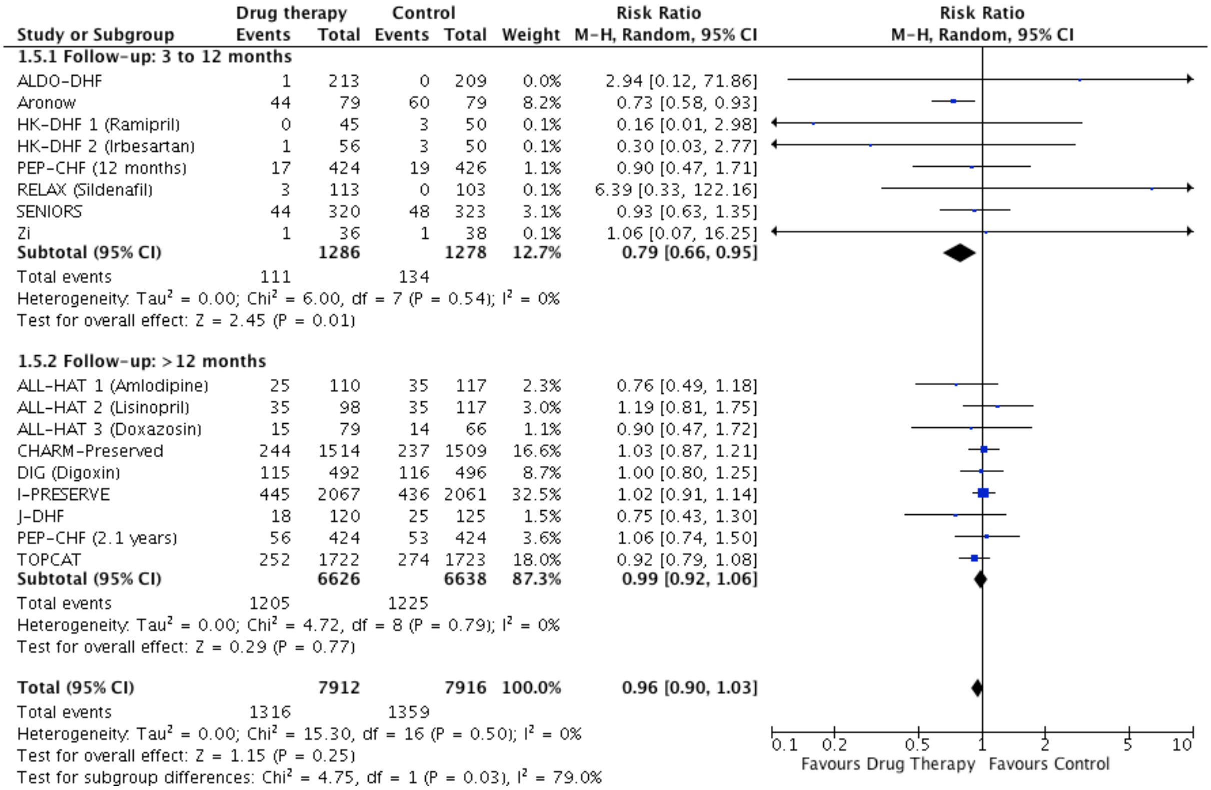

Supplementary Figure 1E: Pooled and individual estimates of relative risk (RR) and 95% CI of the primary outcome all cause-mortality stratified by mean LV ejection fraction: <60% and ≥60%. Random effects model used.

Study or Subgroup	Drug therapy Events	Drug therapy Total	Control Events	Control Total	Weight	Risk Ratio M-H, Random, 95% CI
**1.6.1 Mean LV ejection fraction <60%**						
Aronow	44	79	60	79	11.3%	0.73 [0.58, 0.93]
CHARM-Preserved	244	1514	237	1509	19.2%	1.03 [0.87, 1.21]
DIG (Digoxin)	115	492	116	496	11.9%	1.00 [0.80, 1.25]
TOPCAT	252	1722	274	1723	20.3%	0.92 [0.79, 1.08]
Zi	1	36	1	38	0.1%	1.06 [0.07, 16.25]
**Subtotal (95% CI)**		**3843**		**3845**	**62.8%**	**0.93 [0.82, 1.05]**
Total events	656		688			
Heterogeneity: $Tau^2 = 0.01$; $Chi^2 = 6.04$, df = 4 (P = 0.20); $I^2 = 34\%$						
Test for overall effect: Z = 1.24 (P = 0.22)						
**1.6.2 Mean LV ejection fraction ≥60%**						
ALDO-DHF	1	213	0	209	0.1%	2.94 [0.12, 71.86]
HK-DHF 1 (Ramipril)	0	45	3	50	0.1%	0.16 [0.01, 2.98]
HK-DHF 2 (Irbesartan)	1	56	3	50	0.2%	0.30 [0.03, 2.77]
I-PRESERVE	445	2067	436	2061	28.9%	1.02 [0.91, 1.14]
J-DHF	18	120	25	125	2.4%	0.75 [0.43, 1.30]
PEP-CHF	56	424	53	426	5.6%	1.06 [0.75, 1.51]
RELAX (Sildenafil)	3	113	0	103	0.1%	6.39 [0.33, 122.16]
**Subtotal (95% CI)**		**3038**		**3024**	**37.2%**	**1.01 [0.90, 1.12]**
Total events	524		520			
Heterogeneity: $Tau^2 = 0.00$; $Chi^2 = 5.82$, df = 6 (P = 0.44); $I^2 = 0\%$						
Test for overall effect: Z = 0.14 (P = 0.89)						
**Total (95% CI)**		**6881**		**6869**	**100.0%**	**0.95 [0.88, 1.04]**
Total events	1180		1208			
Heterogeneity: $Tau^2 = 0.00$; $Chi^2 = 12.93$, df = 11 (P = 0.30); $I^2 = 15\%$						
Test for overall effect: Z = 1.05 (P = 0.29)						
Test for subgroup differences: $Chi^2 = 1.05$, df = 1 (P = 0.31), $I^2 = 4.9\%$						

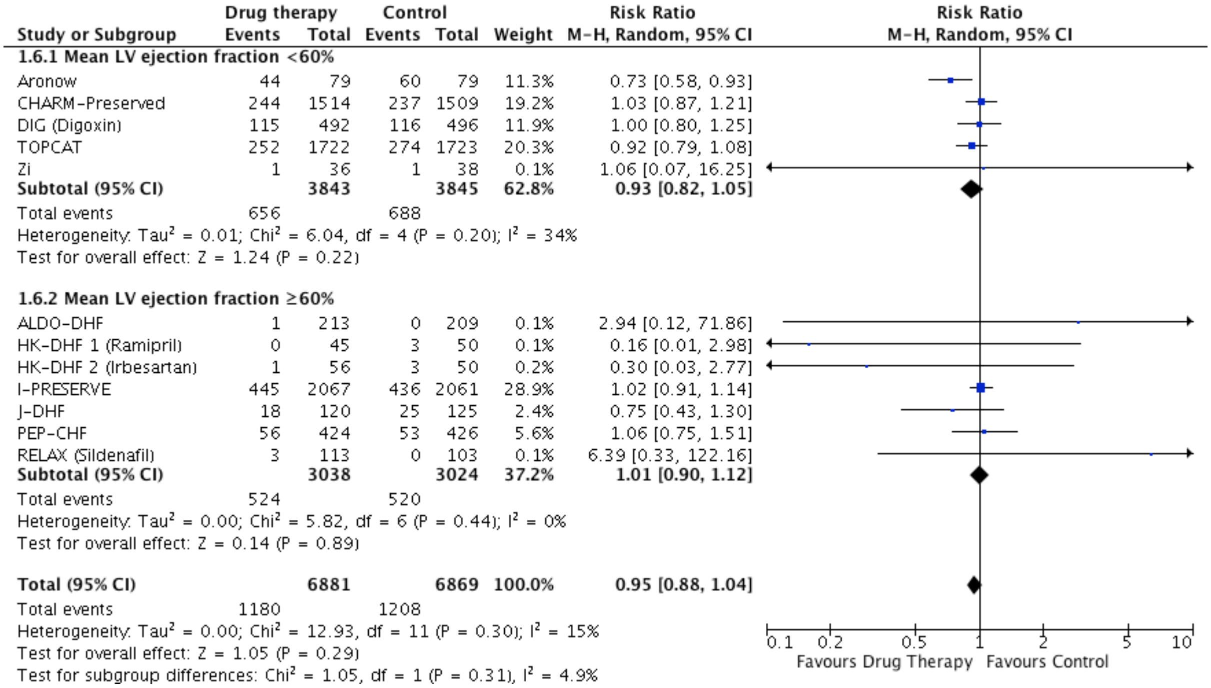

Supplementary Figure 2A: Pooled and individual estimates of relative risk (RR) and 95% CI of the cardiovascular mortality for different drug classes. Data is shown stratified by beta-blockers, RAAS antagonists (which includes ACE inhibitors, ARB, and MRA), and other drug classes. Random effects model used.

Study or Subgroup	Drug therapy Events	Drug therapy Total	Control Events	Control Total	Weight	Risk Ratio M-H, Random, 95% CI
**2.2.1 Beta-blocker**						
Aronow	40	79	56	79	11.0%	0.71 [0.55, 0.93]
J-DHF	8	120	7	125	0.8%	1.19 [0.45, 3.18]
SENIORS	28	320	35	323	3.3%	0.81 [0.50, 1.29]
**Subtotal (95% CI)**		**519**		**527**	**15.1%**	**0.75 [0.60, 0.94]**
Total events	76		98			
Heterogeneity: Tau² = 0.00; Chi² = 1.18, df = 2 (P = 0.55); I² = 0%						
Test for overall effect: Z = 2.51 (P = 0.01)						
**2.2.2 Renin-angiotensin-aldosterone system blockade**						
CHARM-Preserved	170	1514	170	1509	18.5%	1.00 [0.82, 1.22]
HK-DHF 1 (Ramipril)	0	45	1	50	0.1%	0.37 [0.02, 8.85]
HK-DHF 2 (Irbesartan)	1	56	1	50	0.1%	0.89 [0.06, 13.90]
I-PRESERVE	311	2067	302	2061	34.7%	1.03 [0.89, 1.19]
PEP-CHF	38	424	40	426	4.1%	0.95 [0.63, 1.46]
TOPCAT	160	1722	176	1723	17.9%	0.91 [0.74, 1.11]
**Subtotal (95% CI)**		**5828**		**5819**	**75.5%**	**0.99 [0.89, 1.09]**
Total events	680		690			
Heterogeneity: Tau² = 0.00; Chi² = 1.31, df = 5 (P = 0.93); I² = 0%						
Test for overall effect: Z = 0.29 (P = 0.77)						
**2.2.3 Others**						
DIG (Digoxin)	81	492	81	496	9.4%	1.01 [0.76, 1.34]
RELAX (Sildenafil)	2	113	0	103	0.1%	4.56 [0.22, 93.91]
**Subtotal (95% CI)**		**605**		**599**	**9.4%**	**1.02 [0.77, 1.35]**
Total events	83		81			
Heterogeneity: Tau² = 0.00; Chi² = 0.95, df = 1 (P = 0.33); I² = 0%						
Test for overall effect: Z = 0.15 (P = 0.88)						
**Total (95% CI)**		**6952**		**6945**	**100.0%**	**0.95 [0.87, 1.03]**
Total events	839		869			
Heterogeneity: Tau² = 0.00; Chi² = 8.47, df = 10 (P = 0.58); I² = 0%						
Test for overall effect: Z = 1.18 (P = 0.24)						
Test for subgroup differences: Chi² = 4.99, df = 2 (P = 0.08), I² = 59.9%						

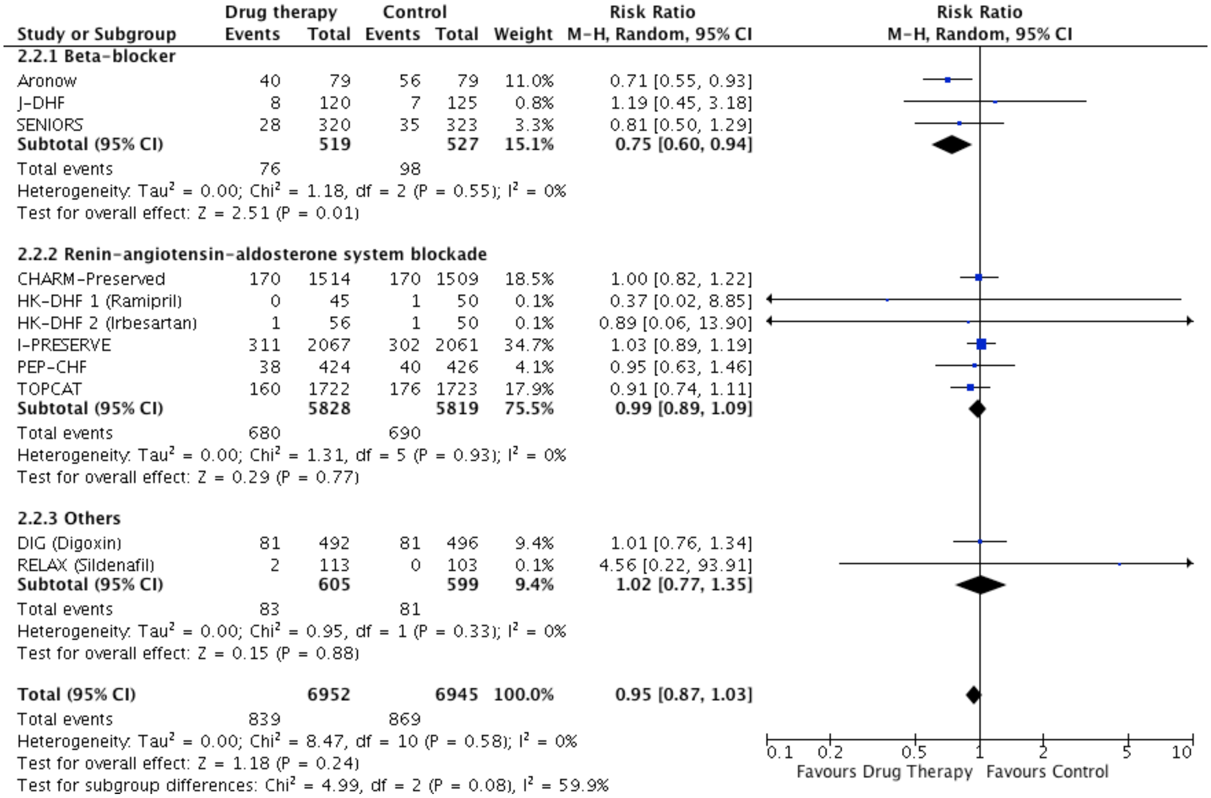

Supplementary Figure 2B: Pooled and individual estimates of risk difference (RD) and 95% CI of cardiovascular mortality for different drug classes. Data is shown stratified by beta-blockers, ACE inhibitors, ARB, and MRA, and other drug classes. Random effects model used.

	Drug therapy		Control			Risk Difference
**Study or Subgroup**	**Events**	**Total**	**Events**	**Total**	**Weight**	**M-H, Random, 95% CI**
**2.3.1 Beta-blocker**						
Aronow	40	79	56	79	0.4%	-0.20 [-0.35, -0.05]
ELANDD	0	57	0	59	7.3%	0.00 [-0.03, 0.03]
J-DHF	8	120	7	125	2.2%	0.01 [-0.05, 0.07]
SENIORS	28	320	35	323	3.8%	-0.02 [-0.07, 0.03]
SWEDIC	0	47	0	50	5.2%	0.00 [-0.04, 0.04]
Takeda	0	19	0	21	0.9%	0.00 [-0.09, 0.09]
**Subtotal (95% CI)**		**642**		**657**	**19.8%**	**-0.01 [-0.05, 0.02]**
Total events	76		98			
Heterogeneity: Tau² = 0.00; Chi² = 14.24, df = 5 (P = 0.01); I² = 65%						
Test for overall effect: Z = 0.69 (P = 0.49)						
**2.3.2 ACE-inhibitor**						
HK-DHF 1 (Ramipril)	0	45	1	50	2.6%	-0.02 [-0.08, 0.04]
Kitzman	0	25	0	34	1.9%	0.00 [-0.07, 0.07]
PEP-CHF	38	424	40	426	5.3%	-0.00 [-0.04, 0.03]
**Subtotal (95% CI)**		**494**		**510**	**9.8%**	**-0.01 [-0.04, 0.02]**
Total events	38		41			
Heterogeneity: Tau² = 0.00; Chi² = 0.30, df = 2 (P = 0.86); I² = 0%						
Test for overall effect: Z = 0.53 (P = 0.60)						
**2.3.3 Angiotensin-receptor blocker**						
CHARM-Preserved	170	1514	170	1509	15.8%	-0.00 [-0.02, 0.02]
HK-DHF 2 (Irbesartan)	1	56	1	50	3.0%	-0.00 [-0.05, 0.05]
I-PRESERVE	311	2067	302	2061	17.1%	0.00 [-0.02, 0.03]
**Subtotal (95% CI)**		**3637**		**3620**	**35.9%**	**0.00 [-0.01, 0.02]**
Total events	482		473			
Heterogeneity: Tau² = 0.00; Chi² = 0.10, df = 2 (P = 0.95); I² = 0%						
Test for overall effect: Z = 0.20 (P = 0.84)						
**2.3.4 Mineralocorticoid-receptor antagonist**						
Kurrelmeyer	0	24	0	24	1.3%	0.00 [-0.08, 0.08]
TOPCAT	160	1722	176	1723	20.5%	-0.01 [-0.03, 0.01]
**Subtotal (95% CI)**		**1746**		**1747**	**21.8%**	**-0.01 [-0.03, 0.01]**
Total events	160		176			
Heterogeneity: Tau² = 0.00; Chi² = 0.05, df = 1 (P = 0.82); I² = 0%						
Test for overall effect: Z = 0.88 (P = 0.38)						
**2.3.5 Others**						
DIG (Digoxin)	81	492	81	496	3.8%	0.00 [-0.04, 0.05]
RELAX (Sildenafil)	2	113	0	103	8.9%	0.02 [-0.01, 0.05]
**Subtotal (95% CI)**		**605**		**599**	**12.7%**	**0.01 [-0.01, 0.04]**
Total events	83		81			
Heterogeneity: Tau² = 0.00; Chi² = 0.79, df = 1 (P = 0.38); I² = 0%						
Test for overall effect: Z = 1.00 (P = 0.32)						
**Total (95% CI)**		**7124**		**7133**	**100.0%**	**-0.00 [-0.01, 0.01]**
Total events	839		869			
Heterogeneity: Tau² = 0.00; Chi² = 11.02, df = 15 (P = 0.75); I² = 0%						
Test for overall effect: Z = 0.38 (P = 0.70)						
Test for subgroup differences: Chi² = 2.49, df = 4 (P = 0.65), I² = 0%						

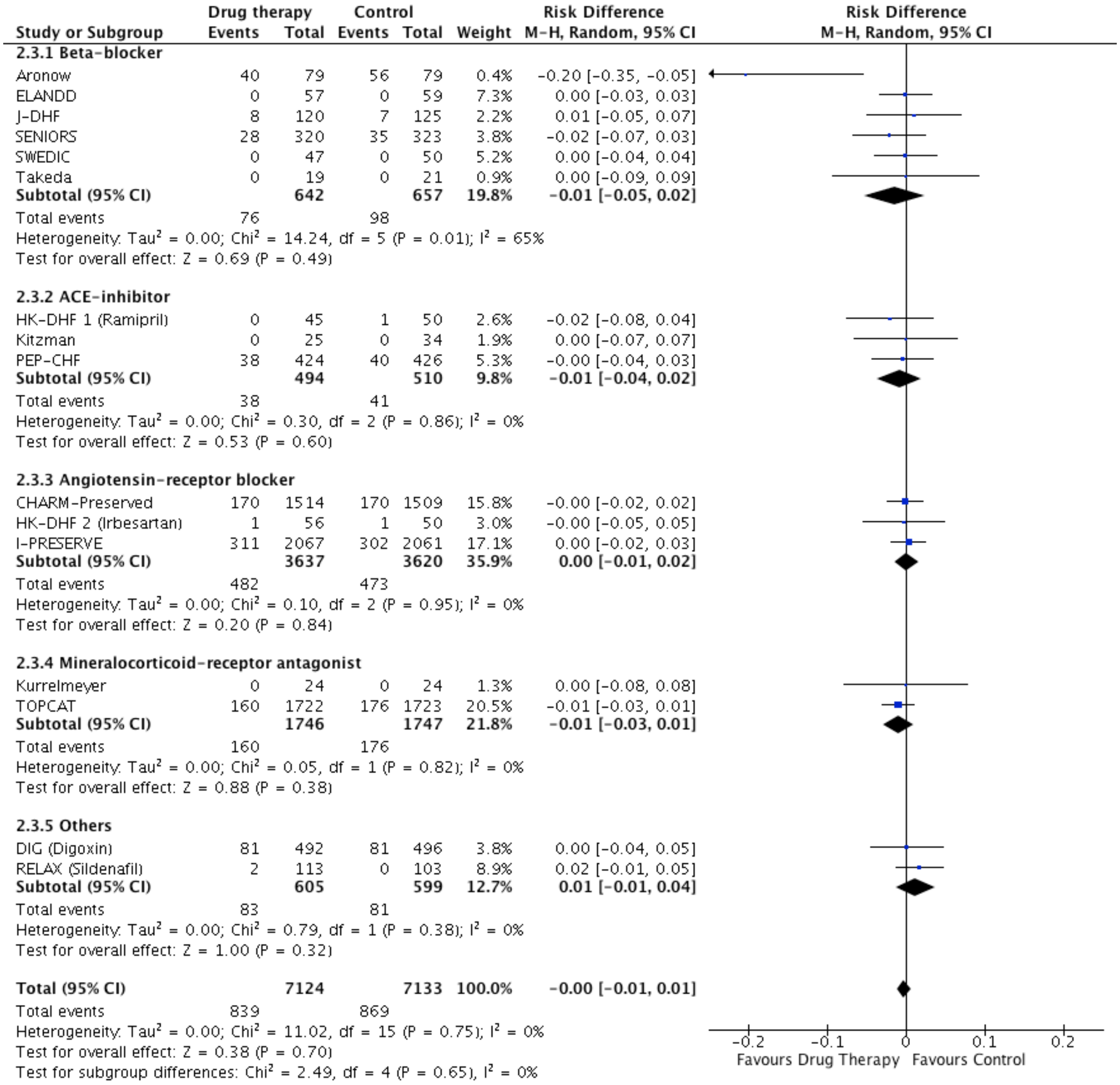

Supplementary Figure 2C: Pooled and individual estimates of relative risk (RR) and 95% CI of cardiovascular mortality stratified by entry LV ejection fraction threshold: 40 to 49% (trials which include patients with HFmrEF), and ≥50% (trials which include only HFpEF). Random effects model used. Only one trial (RELAX) using an LV ejection fraction threshold of 50% reported cardiovascular mortality.

	Drug therapy		Control			Risk Ratio
**Study or Subgroup**	**Events**	**Total**	**Events**	**Total**	**Weight**	**M-H, Random, 95% CI**
**2.4.1 Trial LV ejection fraction entry cut-off: 40 to 49%**						
Aronow	40	79	56	79	11.0%	0.71 [0.55, 0.93]
CHARM-Preserved	170	1514	170	1509	18.5%	1.00 [0.82, 1.22]
DIG (Digoxin)	81	492	81	496	9.4%	1.01 [0.76, 1.34]
HK-DHF 1 (Ramipril)	0	45	1	50	0.1%	0.37 [0.02, 8.85]
HK-DHF 2 (Irbesartan)	1	56	1	50	0.1%	0.89 [0.06, 13.90]
I-PRESERVE	311	2067	302	2061	34.7%	1.03 [0.89, 1.19]
J-DHF	8	120	7	125	0.8%	1.19 [0.45, 3.18]
PEP-CHF	38	424	40	426	4.1%	0.95 [0.63, 1.46]
SENIORS	28	320	35	323	3.3%	0.81 [0.50, 1.29]
TOPCAT	160	1722	176	1723	17.9%	0.91 [0.74, 1.11]
**Subtotal (95% CI)**		**6839**		**6842**	**99.9%**	**0.95 [0.87, 1.03]**
Total events	837		869			
Heterogeneity: Tau² = 0.00; Chi² = 7.42, df = 9 (P = 0.59); I² = 0%						
Test for overall effect: Z = 1.21 (P = 0.23)						
**2.4.2 Trial LV ejection fraction entry cut-off: ≥50%**						
RELAX (Sildenafil)	2	113	0	103	0.1%	4.56 [0.22, 93.91]
**Subtotal (95% CI)**		**113**		**103**	**0.1%**	**4.56 [0.22, 93.91]**
Total events	2		0			
Heterogeneity: Not applicable						
Test for overall effect: Z = 0.98 (P = 0.33)						
**Total (95% CI)**		**6952**		**6945**	**100.0%**	**0.95 [0.87, 1.03]**
Total events	839		869			
Heterogeneity: Tau² = 0.00; Chi² = 8.47, df = 10 (P = 0.58); I² = 0%						
Test for overall effect: Z = 1.18 (P = 0.24)						
Test for subgroup differences: Chi² = 1.04, df = 1 (P = 0.31), I² = 3.4%						

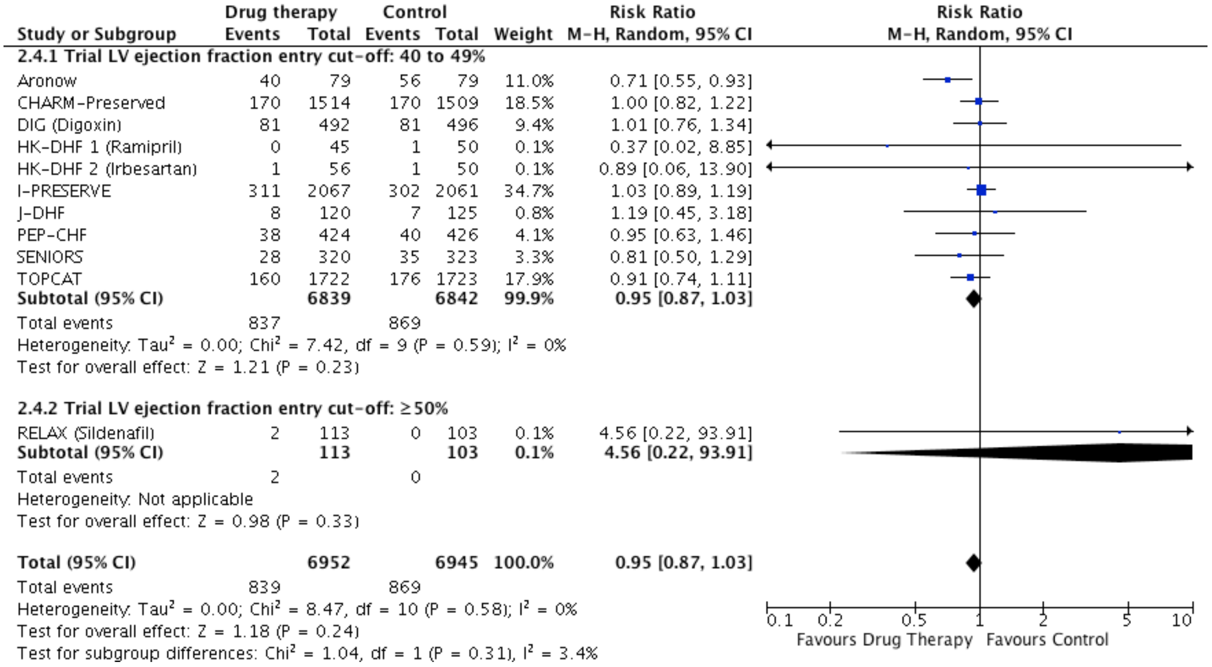

Supplementary Figure 2D: Pooled and individual estimates of relative risk (RR) and 95% CI of cardiovascular mortality stratified by follow-up duration: between 3 to 12 months and greater than 12 months. Random effects model used. PEP-CHF reported outcomes at 12 months follow-up in addition to end of follow-up (mean 2.1 years).

Study or Subgroup	Drug therapy Events	Drug therapy Total	Control Events	Control Total	Weight	Risk Ratio M-H, Random, 95% CI
**2.5.1 Follow-up: 3 to 12 months**						
Aronow	40	79	56	79	10.9%	0.71 [0.55, 0.93]
HK-DHF 1 (Ramipril)	0	45	1	50	0.1%	0.37 [0.02, 8.85]
HK-DHF 2 (Irbesartan)	1	56	1	50	0.1%	0.89 [0.06, 13.90]
PEP-CHF (12 months)	10	424	17	426	1.2%	0.59 [0.27, 1.28]
RELAX (Sildenafil)	2	113	0	103	0.1%	4.56 [0.22, 93.91]
**Subtotal (95% CI)**		**717**		**708**	**12.4%**	**0.71 [0.55, 0.90]**
Total events	53		75			
Heterogeneity: Tau² = 0.00; Chi² = 1.86, df = 4 (P = 0.76); I² = 0%						
Test for overall effect: Z = 2.78 (P = 0.005)						
**2.5.2 Follow-up: >12 months**						
CHARM-Preserved	170	1514	170	1509	18.3%	1.00 [0.82, 1.22]
DIG (Digoxin)	81	492	81	496	9.2%	1.01 [0.76, 1.34]
I-PRESERVE	311	2067	302	2061	34.3%	1.03 [0.89, 1.19]
J-DHF	8	120	7	125	0.8%	1.19 [0.45, 3.18]
PEP-CHF (2.1 years)	38	424	40	426	4.1%	0.95 [0.63, 1.46]
SENIORS	28	320	35	353	3.3%	0.88 [0.55, 1.42]
TOPCAT	160	1722	176	1723	17.7%	0.91 [0.74, 1.11]
**Subtotal (95% CI)**		**6659**		**6693**	**87.6%**	**0.99 [0.90, 1.08]**
Total events	796		811			
Heterogeneity: Tau² = 0.00; Chi² = 1.31, df = 6 (P = 0.97); I² = 0%						
Test for overall effect: Z = 0.30 (P = 0.76)						
**Total (95% CI)**		**7376**		**7401**	**100.0%**	**0.95 [0.87, 1.03]**
Total events	849		886			
Heterogeneity: Tau² = 0.00; Chi² = 9.54, df = 11 (P = 0.57); I² = 0%						
Test for overall effect: Z = 1.26 (P = 0.21)						
Test for subgroup differences: Chi² = 6.25, df = 1 (P = 0.01), I² = 84.0%						

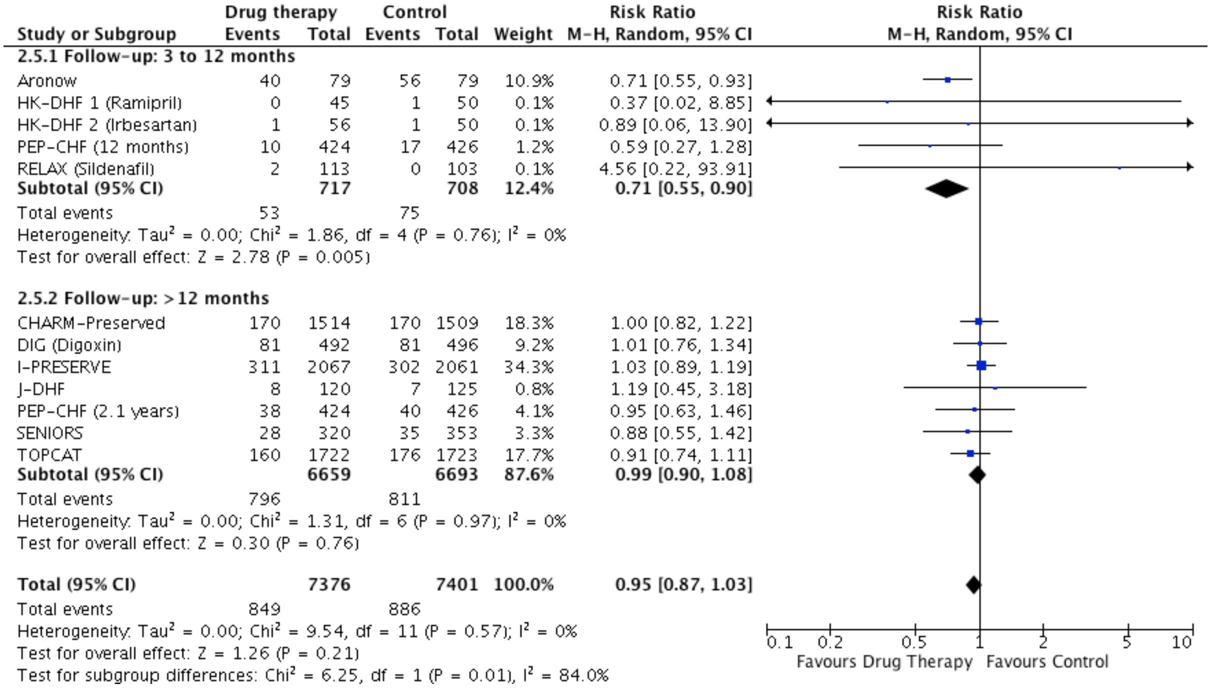

Supplementary Figure 2E: Pooled and individual estimates of relative risk (RR) and 95% CI of cardiovascular mortality stratified by mean LV ejection fraction: <60% and ≥60%. Random effects model used.

Study or Subgroup	Drug therapy Events	Drug therapy Total	Control Events	Control Total	Weight	Risk Ratio M-H, Random, 95% CI
**2.6.1 Mean LV ejection fraction <60%**						
Aronow	40	79	56	79	11.4%	0.71 [0.55, 0.93]
CHARM-Preserved	170	1514	170	1509	19.1%	1.00 [0.82, 1.22]
DIG (Digoxin)	81	492	81	496	9.7%	1.01 [0.76, 1.34]
TOPCAT	160	1722	176	1723	18.5%	0.91 [0.74, 1.11]
**Subtotal (95% CI)**		**3807**		**3807**	**58.7%**	**0.90 [0.78, 1.05]**
Total events	451		483			
Heterogeneity: Tau² = 0.01; Chi² = 4.85, df = 3 (P = 0.18); I² = 38%						
Test for overall effect: Z = 1.33 (P = 0.18)						
**2.6.2 Mean LV ejection fraction ≥60%**						
HK-DHF 1 (Ramipril)	0	45	1	50	0.1%	0.37 [0.02, 8.85]
HK-DHF 2 (Irbesartan)	1	56	1	50	0.1%	0.89 [0.06, 13.90]
I-PRESERVE	311	2067	302	2061	35.9%	1.03 [0.89, 1.19]
J-DHF	8	120	7	125	0.8%	1.19 [0.45, 3.18]
PEP-CHF	38	424	40	426	4.3%	0.95 [0.63, 1.46]
RELAX (Sildenafil)	2	113	0	103	0.1%	4.56 [0.22, 93.91]
**Subtotal (95% CI)**		**2825**		**2815**	**41.3%**	**1.02 [0.89, 1.17]**
Total events	360		351			
Heterogeneity: Tau² = 0.00; Chi² = 1.54, df = 5 (P = 0.91); I² = 0%						
Test for overall effect: Z = 0.32 (P = 0.75)						
**Total (95% CI)**		**6632**		**6622**	**100.0%**	**0.95 [0.87, 1.04]**
Total events	811		834			
Heterogeneity: Tau² = 0.00; Chi² = 8.02, df = 9 (P = 0.53); I² = 0%						
Test for overall effect: Z = 1.04 (P = 0.30)						
Test for subgroup differences: Chi² = 1.43, df = 1 (P = 0.23), I² = 30.0%						

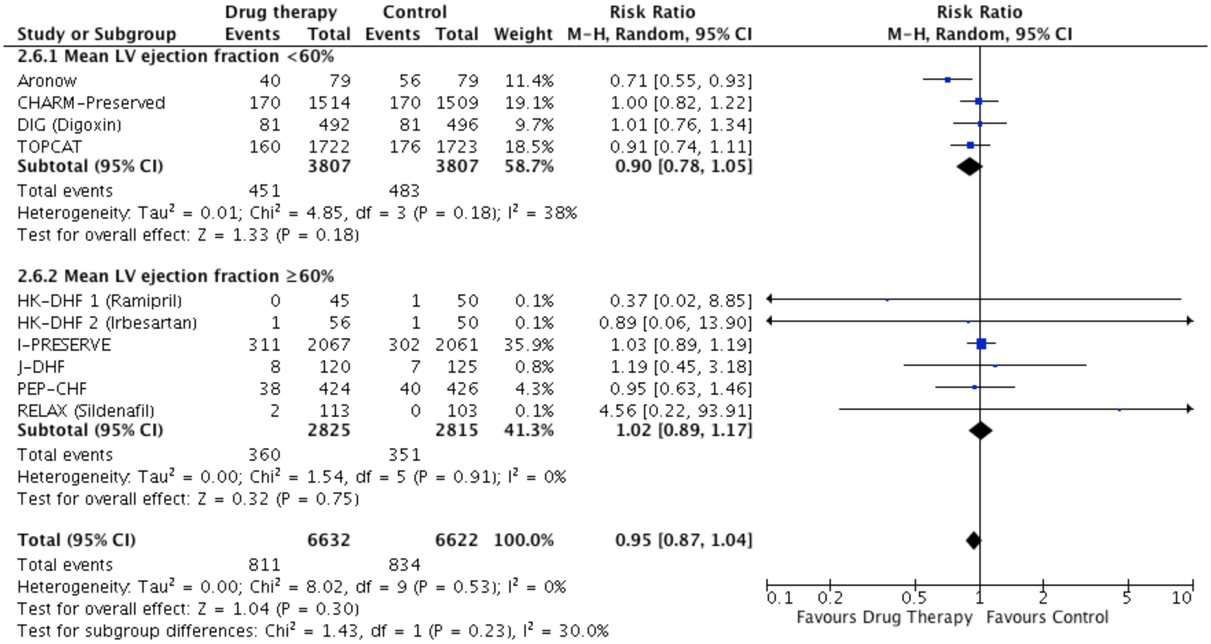

Supplementary Figure 3A: Pooled and individual estimates of relative risk (RR) and 95% CI of heart failure hospitalisation for different drug classes. Data is shown stratified by beta-blockers, RAAS antagonists (which includes ACE inhibitors, ARB, and MRA), and other drug classes. Random effects model used.

Study or Subgroup	Drug therapy Events	Drug therapy Total	Control Events	Control Total	Weight	Risk Ratio M-H, Random, 95% CI
**3.2.1 Beta-blocker**						
J-DHF	21	120	34	125	2.7%	0.64 [0.40, 1.04]
SWEDIC	2	47	0	50	0.1%	5.31 [0.26, 107.85]
Takeda	1	19	2	21	0.1%	0.55 [0.05, 5.62]
**Subtotal (95% CI)**		**186**		**196**	**2.9%**	**0.67 [0.42, 1.07]**
Total events	24		36			
Heterogeneity: Tau² = 0.00; Chi² = 1.90, df = 2 (P = 0.39); I² = 0%						
Test for overall effect: Z = 1.66 (P = 0.10)						
**3.2.2 Renin-angiotensin-aldosterone system blockade**						
CHARM-Preserved	241	1514	276	1509	25.4%	0.87 [0.74, 1.02]
HK-DHF 1 (Ramipril)	5	45	6	50	0.5%	0.93 [0.30, 2.83]
HK-DHF 2 (Irbesartan)	6	56	6	50	0.6%	0.89 [0.31, 2.59]
I-PRESERVE	325	2061	336	2067	32.2%	0.97 [0.84, 1.12]
PEP-CHF	64	424	73	426	6.7%	0.88 [0.65, 1.20]
RAAM-PEF	1	21	2	23	0.1%	0.55 [0.05, 5.61]
TOPCAT	206	1722	245	1723	21.1%	0.84 [0.71, 1.00]
Zi	2	36	5	38	0.3%	0.42 [0.09, 2.04]
**Subtotal (95% CI)**		**5879**		**5886**	**86.7%**	**0.90 [0.82, 0.98]**
Total events	850		949			
Heterogeneity: Tau² = 0.00; Chi² = 2.95, df = 7 (P = 0.89); I² = 0%						
Test for overall effect: Z = 2.49 (P = 0.01)						
**3.2.3 Others**						
DIG (Digoxin)	89	492	108	496	10.0%	0.83 [0.65, 1.07]
Zile (Sitaxsentan)	5	128	5	64	0.4%	0.50 [0.15, 1.66]
**Subtotal (95% CI)**		**620**		**560**	**10.4%**	**0.81 [0.64, 1.04]**
Total events	94		113			
Heterogeneity: Tau² = 0.00; Chi² = 0.66, df = 1 (P = 0.42); I² = 0%						
Test for overall effect: Z = 1.65 (P = 0.10)						
**Total (95% CI)**		**6685**		**6642**	**100.0%**	**0.88 [0.81, 0.95]**
Total events	968		1098			
Heterogeneity: Tau² = 0.00; Chi² = 7.34, df = 12 (P = 0.83); I² = 0%						
Test for overall effect: Z = 3.13 (P = 0.002)						
Test for subgroup differences: Chi² = 1.87, df = 2 (P = 0.39), I² = 0%						

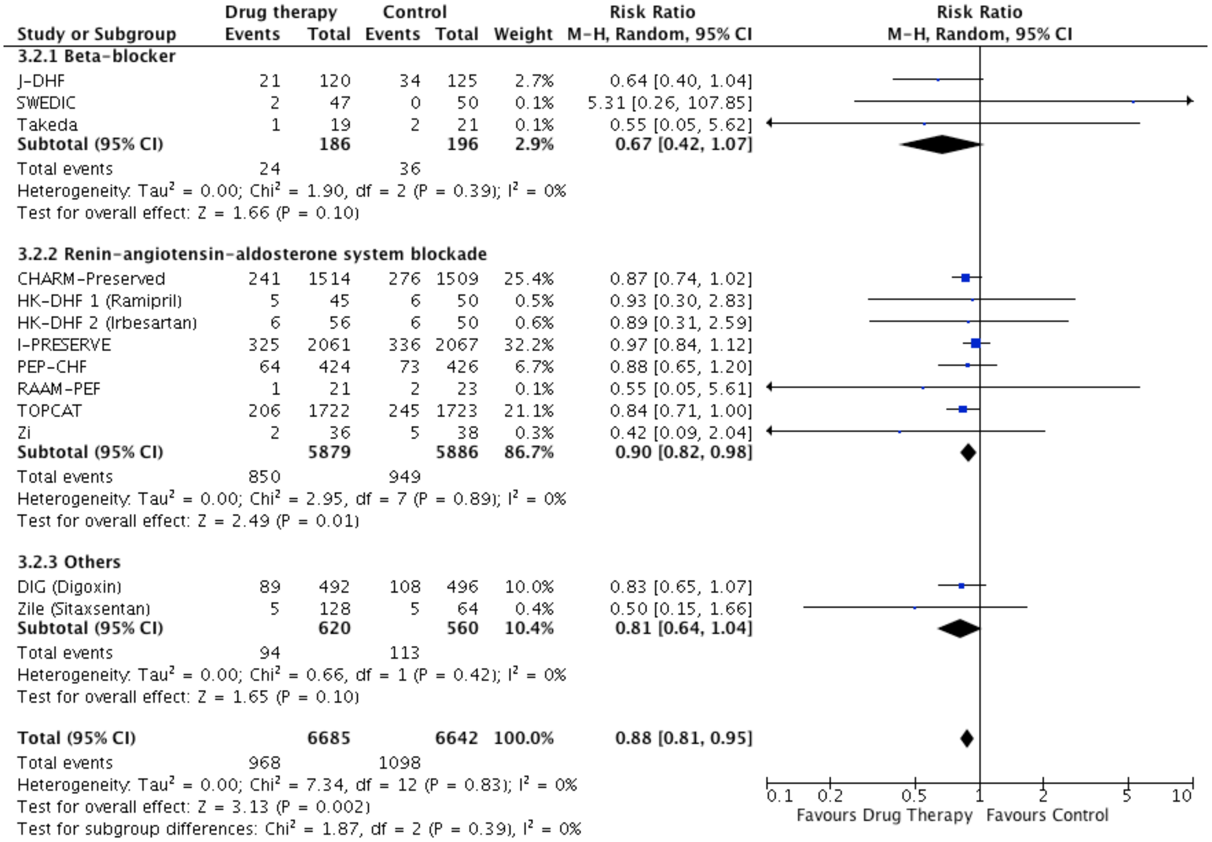

Supplementary Figure 3B: Pooled and individual estimates of risk difference (RD) and 95% CI of heart failure hospitalisation for different drug classes. Data is shown stratified by beta-blockers, ACE inhibitors, ARB, and MRA, and other drug classes. Random effects model used.

Study or Subgroup	Drug therapy Events	Drug therapy Total	Control Events	Control Total	Weight	Risk Difference M-H, Random, 95% CI
**3.3.1 Beta-blocker**						
ELANDD	0	57	0	59	11.4%	0.00 [-0.03, 0.03]
J-DHF	21	120	34	125	1.2%	-0.10 [-0.20, 0.01]
SWEDIC	2	47	0	50	2.7%	0.04 [-0.03, 0.11]
Takeda	1	19	2	21	0.5%	-0.04 [-0.20, 0.12]
**Subtotal (95% CI)**		**243**		**255**	**15.7%**	**-0.02 [-0.10, 0.07]**
Total events	24		36			
Heterogeneity: Tau² = 0.00; Chi² = 13.48, df = 3 (P = 0.004); I² = 78%						
Test for overall effect: Z = 0.39 (P = 0.70)						
**3.3.2 ACE-inhibitor**						
HK-DHF 1 (Ramipril)	5	45	6	50	0.8%	-0.01 [-0.14, 0.12]
PEP-CHF	64	424	73	426	5.1%	-0.02 [-0.07, 0.03]
Zi	2	36	5	38	0.7%	-0.08 [-0.21, 0.05]
**Subtotal (95% CI)**		**505**		**514**	**6.6%**	**-0.03 [-0.07, 0.02]**
Total events	71		84			
Heterogeneity: Tau² = 0.00; Chi² = 0.68, df = 2 (P = 0.71); I² = 0%						
Test for overall effect: Z = 1.14 (P = 0.26)						
**3.3.3 Angiotensin-receptor blocker**						
CHARM-Preserved	241	1514	276	1509	17.3%	-0.02 [-0.05, 0.00]
HK-DHF 2 (Irbesartan)	6	56	6	50	0.9%	-0.01 [-0.13, 0.11]
I-PRESERVE	325	2061	336	2067	24.9%	-0.00 [-0.03, 0.02]
RAAM-PEF	1	21	2	23	0.6%	-0.04 [-0.19, 0.11]
**Subtotal (95% CI)**		**3652**		**3649**	**43.7%**	**-0.01 [-0.03, 0.00]**
Total events	573		620			
Heterogeneity: Tau² = 0.00; Chi² = 1.24, df = 3 (P = 0.74); I² = 0%						
Test for overall effect: Z = 1.50 (P = 0.13)						
**3.3.4 Mineralocorticoid-receptor antagonist**						
Kurrelmeyer	0	24	0	24	2.1%	0.00 [-0.08, 0.08]
TOPCAT	206	1722	245	1723	24.6%	-0.02 [-0.05, -0.00]
**Subtotal (95% CI)**		**1746**		**1747**	**26.7%**	**-0.02 [-0.04, 0.00]**
Total events	206		245			
Heterogeneity: Tau² = 0.00; Chi² = 0.32, df = 1 (P = 0.57); I² = 0%						
Test for overall effect: Z = 1.89 (P = 0.06)						
**3.3.5 Others**						
DIG (Digoxin)	89	492	108	496	5.0%	-0.04 [-0.09, 0.01]
Zile (Sitaxsentan)	5	128	5	64	2.3%	-0.04 [-0.11, 0.03]
**Subtotal (95% CI)**		**620**		**560**	**7.3%**	**-0.04 [-0.08, 0.00]**
Total events	94		113			
Heterogeneity: Tau² = 0.00; Chi² = 0.00, df = 1 (P = 0.96); I² = 0%						
Test for overall effect: Z = 1.78 (P = 0.07)						
**Total (95% CI)**		**6766**		**6725**	**100.0%**	**-0.02 [-0.03, -0.00]**
Total events	968		1098			
Heterogeneity: Tau² = 0.00; Chi² = 10.31, df = 14 (P = 0.74); I² = 0%						
Test for overall effect: Z = 2.78 (P = 0.005)						
Test for subgroup differences: Chi² = 1.37, df = 4 (P = 0.85), I² = 0%						

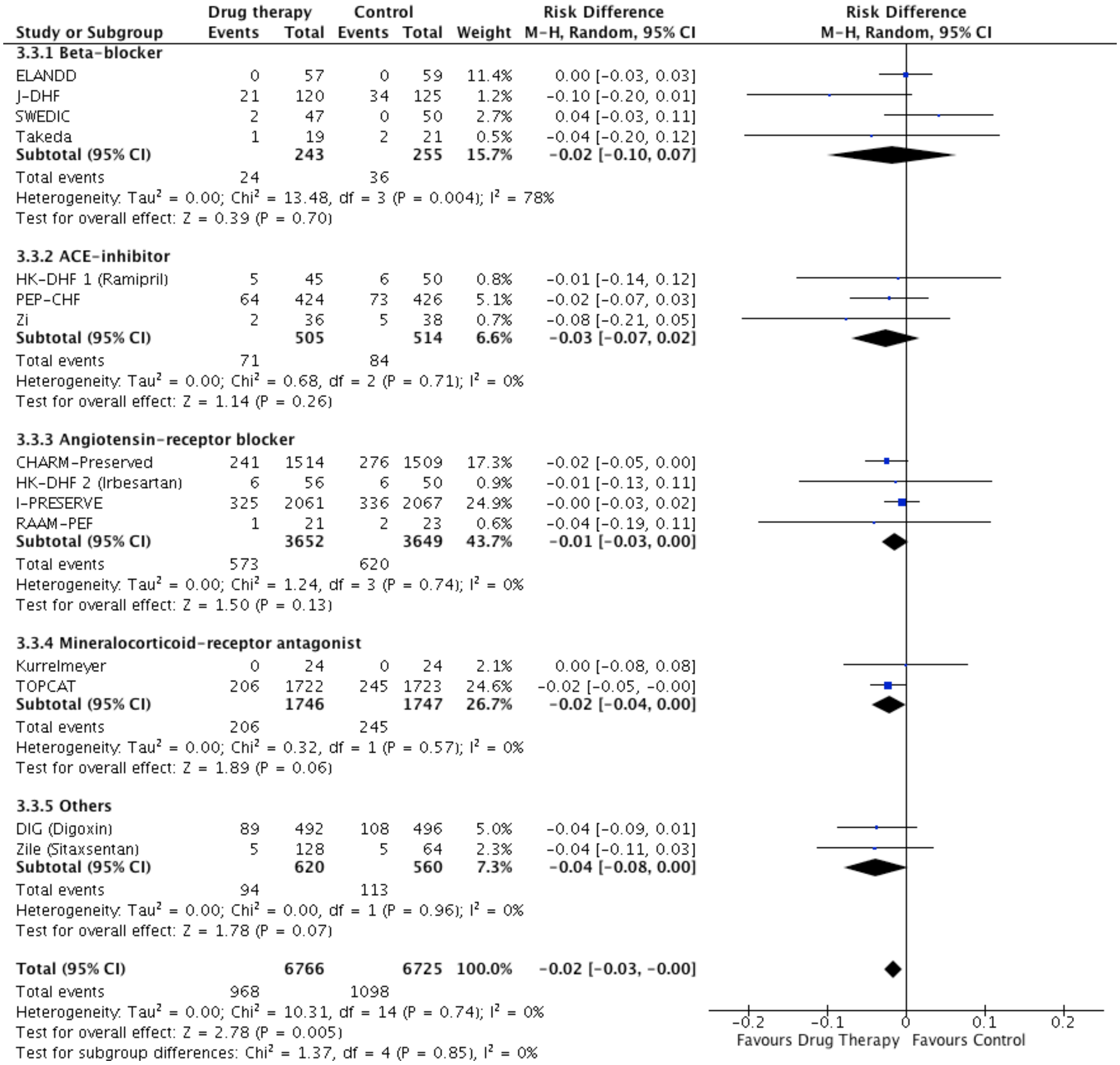

Supplementary Figure 3C: Pooled and individual estimates of relative risk (RR) and 95% CI of heart failure hospitalisation stratified by entry LV ejection fraction threshold: 40 to 49% (trials which include patients with HFmrEF), and ≥50% (trials which include only HFpEF). Random effects model used.

Study or Subgroup	Drug therapy Events	Drug therapy Total	Control Events	Control Total	Weight	Risk Ratio M-H, Random, 95% CI
**3.4.1 Trial LV ejection fraction entry cut-off: 40 to 49%**						
CHARM-Preserved	241	1514	276	1509	25.4%	0.87 [0.74, 1.02]
DIG (Digoxin)	89	492	108	496	10.0%	0.83 [0.65, 1.07]
HK-DHF 1 (Ramipril)	5	45	6	50	0.5%	0.93 [0.30, 2.83]
HK-DHF 2 (Irbesartan)	6	56	6	50	0.6%	0.89 [0.31, 2.59]
I-PRESERVE	325	2061	336	2067	32.2%	0.97 [0.84, 1.12]
J-DHF	21	120	34	125	2.7%	0.64 [0.40, 1.04]
PEP-CHF	64	424	73	426	6.7%	0.88 [0.65, 1.20]
SWEDIC	2	47	0	50	0.1%	5.31 [0.26, 107.85]
Takeda	1	19	2	21	0.1%	0.55 [0.05, 5.62]
TOPCAT	206	1722	245	1723	21.1%	0.84 [0.71, 1.00]
Zi	2	36	5	38	0.3%	0.42 [0.09, 2.04]
**Subtotal (95% CI)**		**6536**		**6555**	**99.4%**	**0.88 [0.82, 0.96]**
Total events	962		1091			
Heterogeneity: $Tau^2 = 0.00$; $Chi^2 = 6.32$, df = 10 (P = 0.79); $I^2 = 0\%$						
Test for overall effect: Z = 3.05 (P = 0.002)						
**3.4.2 Trial LV ejection fraction entry cut-off: ≥50%**						
RAAM-PEF	1	21	2	23	0.1%	0.55 [0.05, 5.61]
Zile (Sitaxsentan)	5	128	5	64	0.4%	0.50 [0.15, 1.66]
**Subtotal (95% CI)**		**149**		**87**	**0.6%**	**0.51 [0.18, 1.48]**
Total events	6		7			
Heterogeneity: $Tau^2 = 0.00$; $Chi^2 = 0.00$, df = 1 (P = 0.95); $I^2 = 0\%$						
Test for overall effect: Z = 1.24 (P = 0.22)						
**Total (95% CI)**		**6685**		**6642**	**100.0%**	**0.88 [0.81, 0.95]**
Total events	968		1098			
Heterogeneity: $Tau^2 = 0.00$; $Chi^2 = 7.34$, df = 12 (P = 0.83); $I^2 = 0\%$						
Test for overall effect: Z = 3.13 (P = 0.002)						
Test for subgroup differences: $Chi^2 = 1.01$, df = 1 (P = 0.31), $I^2 = 1.3\%$						

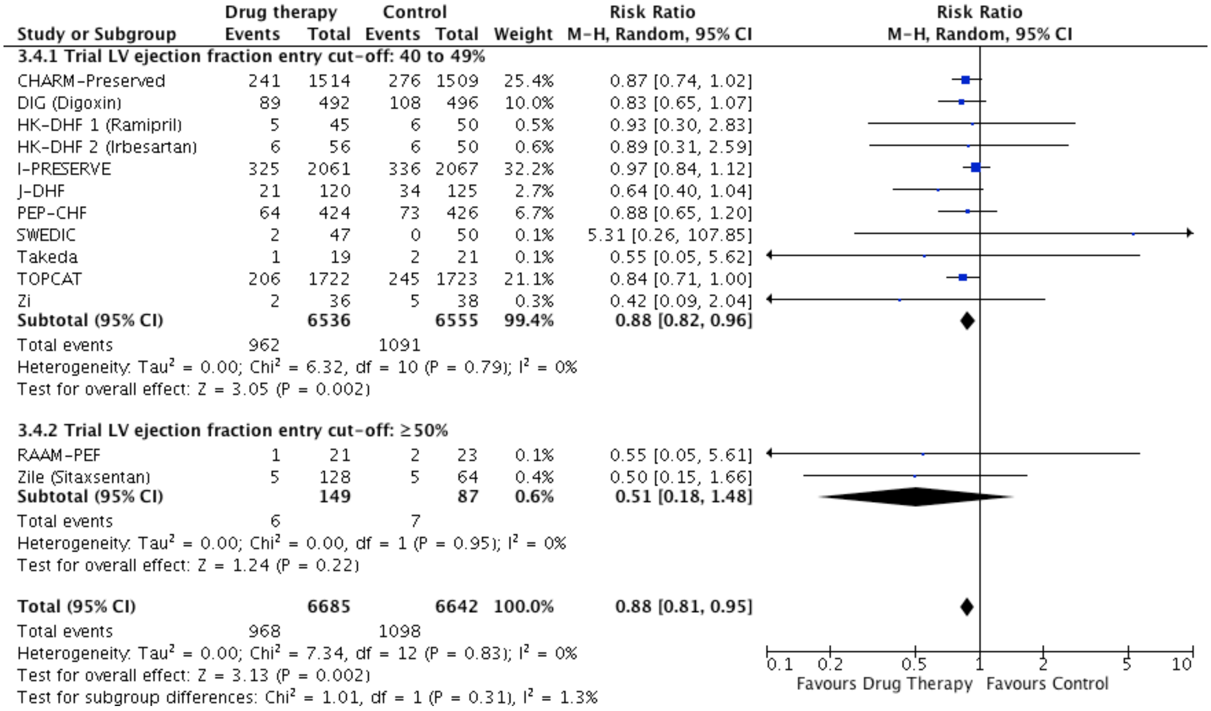

Supplementary Figure 3D: Pooled and individual estimates of relative risk (RR) and 95% CI of heart failure hospitalisation stratified by follow-up duration: between 3 to 12 months and greater than 12 months. Random effects model used. PEP-CHF reported outcomes at 12 months follow-up in addition to end of follow-up (mean 2.1 years).

Study or Subgroup	Drug therapy Events	Drug therapy Total	Control Events	Control Total	Weight	Risk Ratio M-H, Random, 95% CI
**3.5.1 Follow-up: 3 to 12 months**						
HK-DHF 1 (Ramipril)	5	45	6	50	0.6%	0.93 [0.30, 2.83]
HK-DHF 2 (Irbesartan)	6	56	6	50	0.7%	0.89 [0.31, 2.59]
PEP-CHF (12 months)	34	424	53	426	4.5%	0.64 [0.43, 0.97]
RAAM-PEF	1	21	2	23	0.1%	0.55 [0.05, 5.61]
SWEDIC	2	47	0	50	0.1%	5.31 [0.26, 107.85]
Takeda	1	19	2	21	0.1%	0.55 [0.05, 5.62]
Zi	2	36	5	38	0.3%	0.42 [0.09, 2.04]
Zile (Sitaxsentan)	5	128	5	64	0.5%	0.50 [0.15, 1.66]
**Subtotal (95% CI)**		**776**		**722**	**7.0%**	**0.67 [0.48, 0.94]**
Total events	56		79			
Heterogeneity: $Tau^2 = 0.00$; $Chi^2 = 3.07$, df = 7 (P = 0.88); $I^2 = 0\%$						
Test for overall effect: Z = 2.35 (P = 0.02)						
**3.5.2 Follow-up: >12 months**						
CHARM-Preserved	241	1514	276	1509	30.7%	0.87 [0.74, 1.02]
DIG (Digoxin)	89	492	108	496	12.0%	0.83 [0.65, 1.07]
I-PRESERVE	325	2061	336	2067	38.9%	0.97 [0.84, 1.12]
J-DHF	21	120	34	125	3.3%	0.64 [0.40, 1.04]
PEP-CHF (2.1 years)	64	424	73	426	8.0%	0.88 [0.65, 1.20]
TOPCAT	0	0	0	0		Not estimable
**Subtotal (95% CI)**		**4611**		**4623**	**93.0%**	**0.90 [0.82, 0.98]**
Total events	740		827			
Heterogeneity: $Tau^2 = 0.00$; $Chi^2 = 3.54$, df = 4 (P = 0.47); $I^2 = 0\%$						
Test for overall effect: Z = 2.36 (P = 0.02)						
**Total (95% CI)**		**5387**		**5345**	**100.0%**	**0.88 [0.81, 0.96]**
Total events	796		906			
Heterogeneity: $Tau^2 = 0.00$; $Chi^2 = 9.31$, df = 12 (P = 0.68); $I^2 = 0\%$						
Test for overall effect: Z = 2.90 (P = 0.004)						
Test for subgroup differences: $Chi^2 = 2.70$, df = 1 (P = 0.10), $I^2 = 63.0\%$						

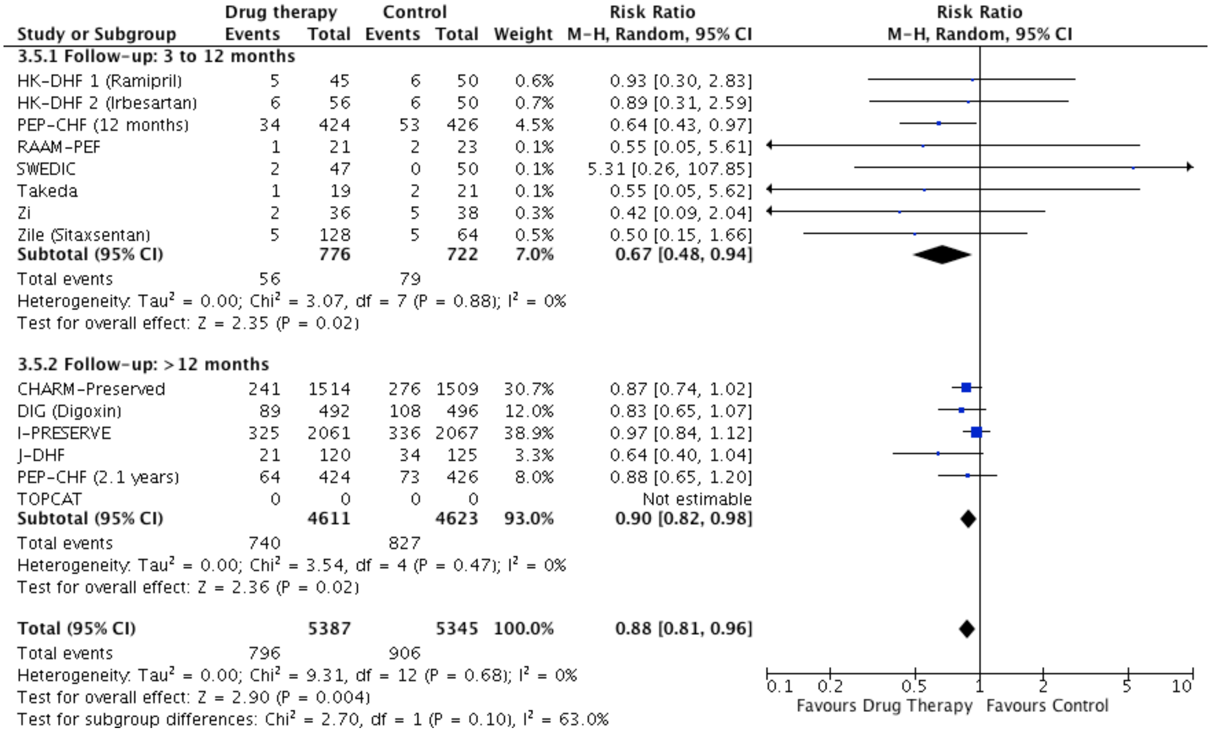

Supplementary Figure 3E: Pooled and individual estimates of relative risk (RR) and 95% CI of heart failure hospitalisation stratified by mean LV ejection fraction: <60% and ≥60%. Random effects model used.

Study or Subgroup	Drug therapy Events	Drug therapy Total	Control Events	Control Total	Weight	Risk Ratio M-H, Random, 95% CI
**3.6.1 Mean LV ejection fraction <60%**						
CHARM-Preserved	241	1514	276	1509	25.4%	0.87 [0.74, 1.02]
DIG (Digoxin)	89	492	108	496	10.0%	0.83 [0.65, 1.07]
Takeda	1	19	2	21	0.1%	0.55 [0.05, 5.62]
TOPCAT	206	1722	245	1723	21.1%	0.84 [0.71, 1.00]
Zi	2	36	5	38	0.3%	0.42 [0.09, 2.04]
**Subtotal (95% CI)**		**3783**		**3787**	**56.8%**	**0.85 [0.76, 0.94]**
Total events	539		636			
Heterogeneity: $Tau^2 = 0.00$; $Chi^2 = 1.02$, df = 4 (P = 0.91); $I^2 = 0\%$						
Test for overall effect: Z = 3.05 (P = 0.002)						
**3.6.2 Mean LV ejection fraction ≥60%**						
HK-DHF 1 (Ramipril)	5	45	6	50	0.5%	0.93 [0.30, 2.83]
HK-DHF 2 (Irbesartan)	6	56	6	50	0.6%	0.89 [0.31, 2.59]
I-PRESERVE	325	2061	336	2067	32.2%	0.97 [0.84, 1.12]
J-DHF	21	120	34	125	2.7%	0.64 [0.40, 1.04]
PEP-CHF	64	424	73	426	6.7%	0.88 [0.65, 1.20]
RAAM-PEF	1	21	2	23	0.1%	0.55 [0.05, 5.61]
Zile (Sitaxsentan)	5	128	5	64	0.4%	0.50 [0.15, 1.66]
**Subtotal (95% CI)**		**2855**		**2805**	**43.2%**	**0.92 [0.82, 1.04]**
Total events	427		462			
Heterogeneity: $Tau^2 = 0.00$; $Chi^2 = 3.92$, df = 6 (P = 0.69); $I^2 = 0\%$						
Test for overall effect: Z = 1.31 (P = 0.19)						
**Total (95% CI)**		**6638**		**6592**	**100.0%**	**0.88 [0.81, 0.95]**
Total events	966		1098			
Heterogeneity: $Tau^2 = 0.00$; $Chi^2 = 5.97$, df = 11 (P = 0.88); $I^2 = 0\%$						
Test for overall effect: Z = 3.16 (P = 0.002)						
Test for subgroup differences: $Chi^2 = 1.03$, df = 1 (P = 0.31), $I^2 = 3.2\%$						

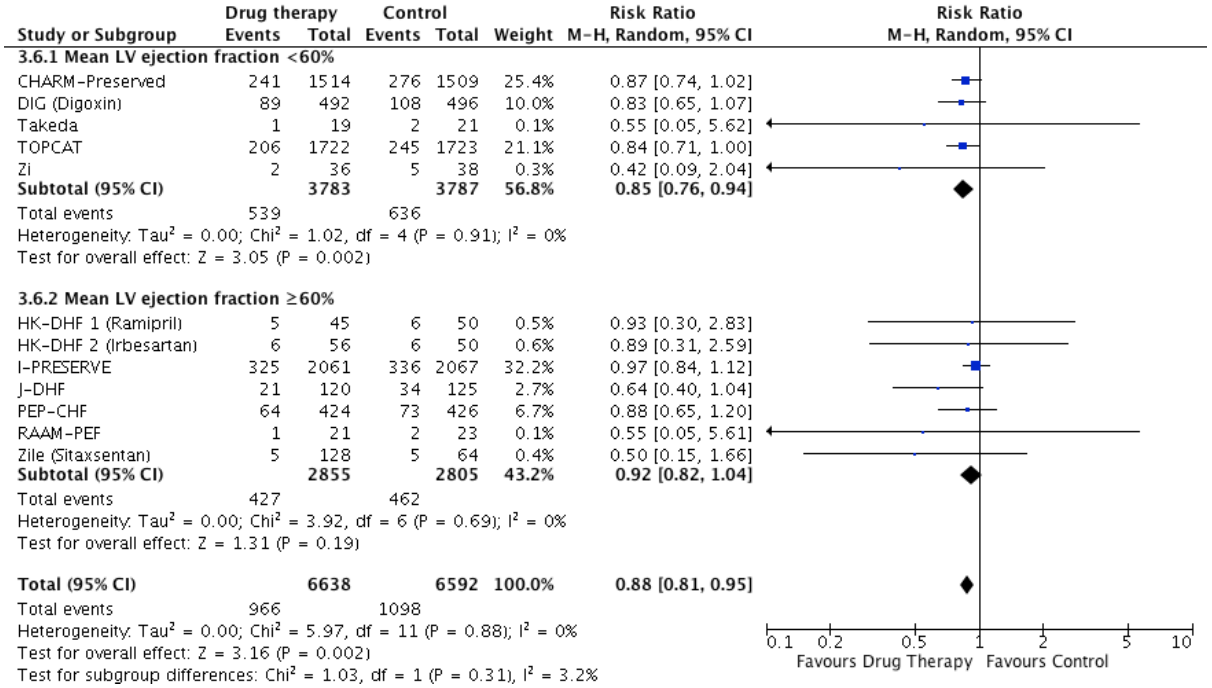

Supplementary Figure 4: Pooled and individual estimates of standardised mean difference (MD) in exercise outcomes: (A) exercise capacity, (B) 6 minutes' walk distance, and (C) VO_2 max. Estimates and 95% CI based on a random effects model. Studies were stratified by individual drug classes (beta-blockers, renin-angiotensin-aldosterone system antagonists and other drug classes). Due to small number of studies, no classification was made by individual drug class blockers. RAAS includes Angiotensin Converting Enzyme inhibitors, Angiotensin Receptor Blockers and Mineralocorticoid Receptor Antagonist.

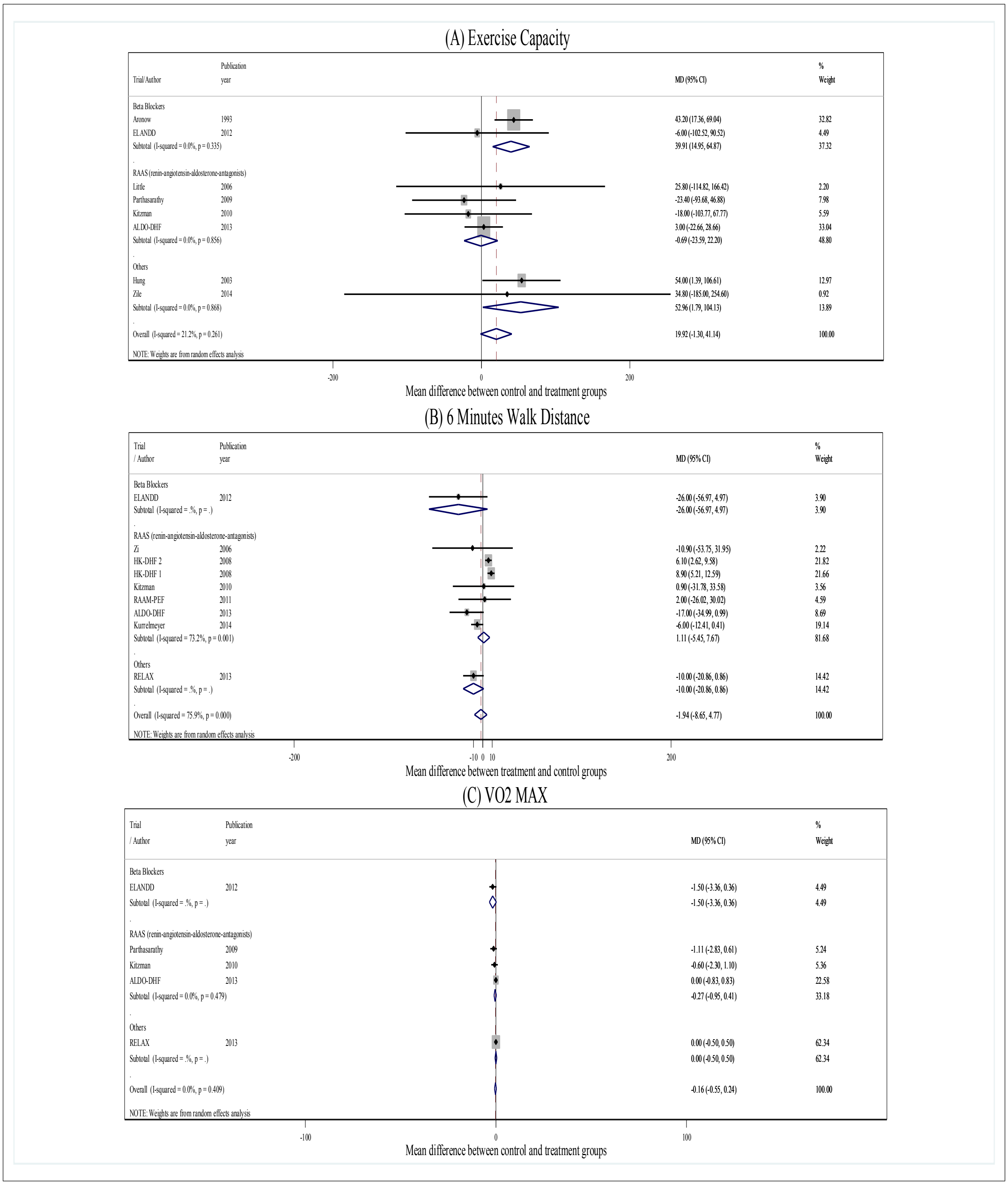

Supplementary Figure 5: Pooled and individual estimates of the standardised mean difference (MD) in quality of life, assessed by Minnesota Living with Heart Failure Questionnaire (MLHFQ). Lower MLHFQ scores associated with reduced symptoms, therefore; negative difference indicates improvement. Data is shown stratified by individual drug classes (beta-blockers, ACE inhibitors, angiotensin receptor blockers, mineralocorticoid receptor antagonists, and other drug classes). Estimates and 95% CI were based on a random effects model.

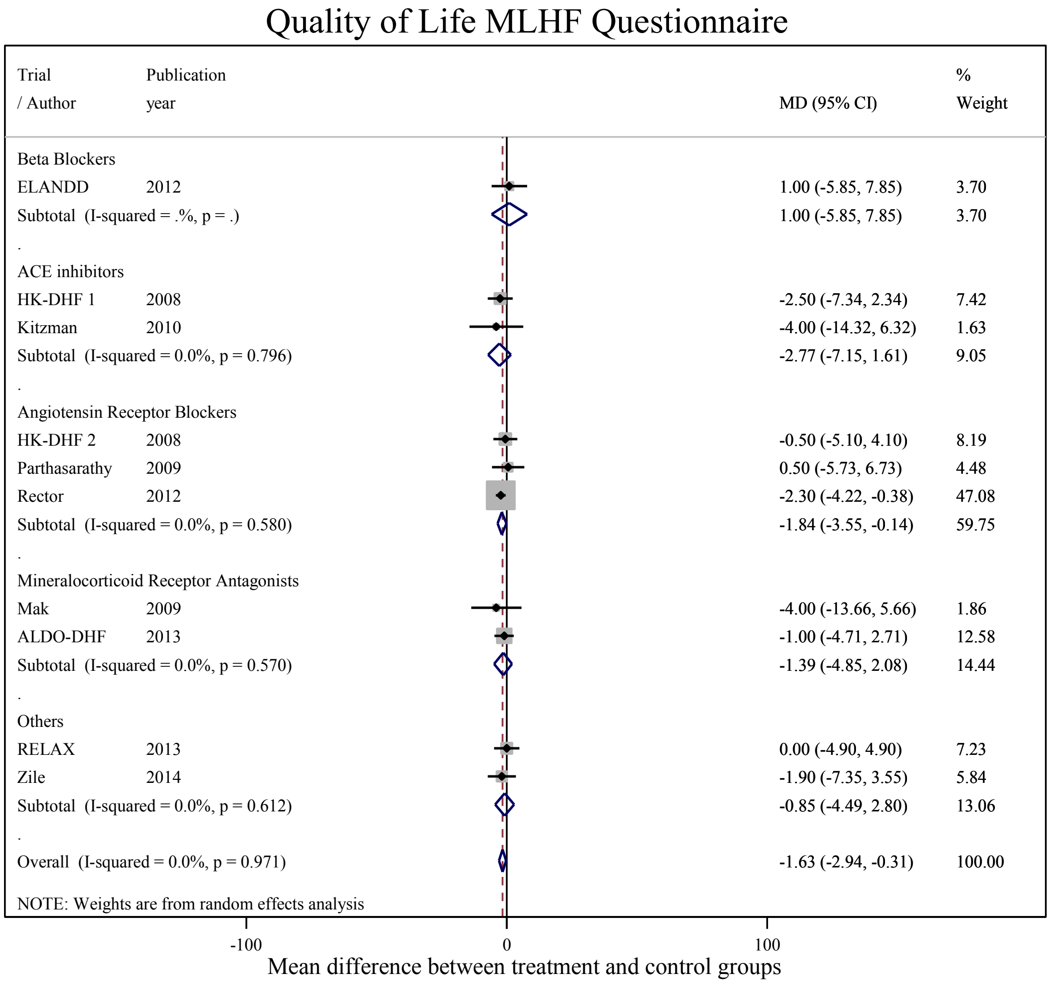

Supplementary Figure 6 – Funnel plot

Supplementary Figure 6A: All-cause mortality

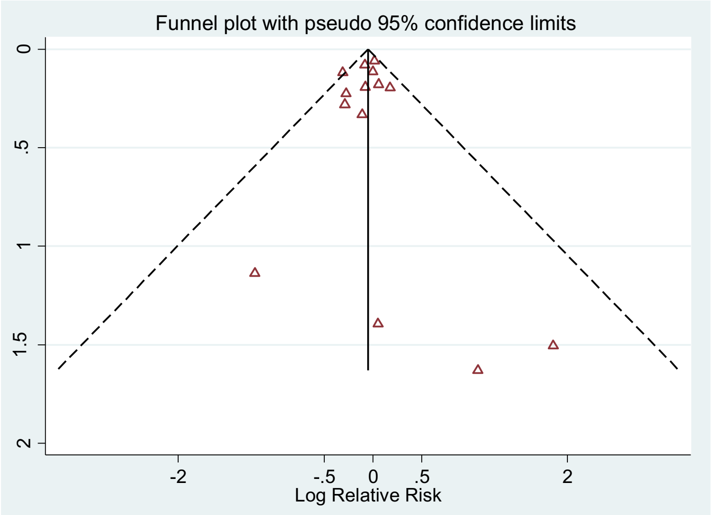

Supplementary Figure 6B: Cardiovascular mortality

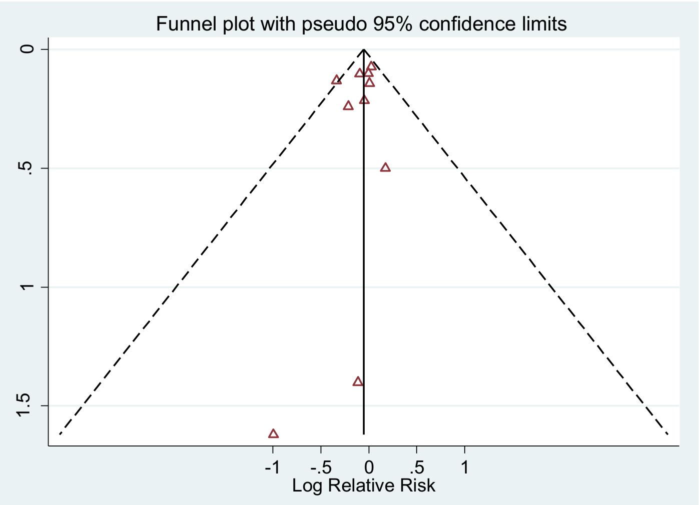

Supplementary Figure 6C: Heart failure hospitalisation

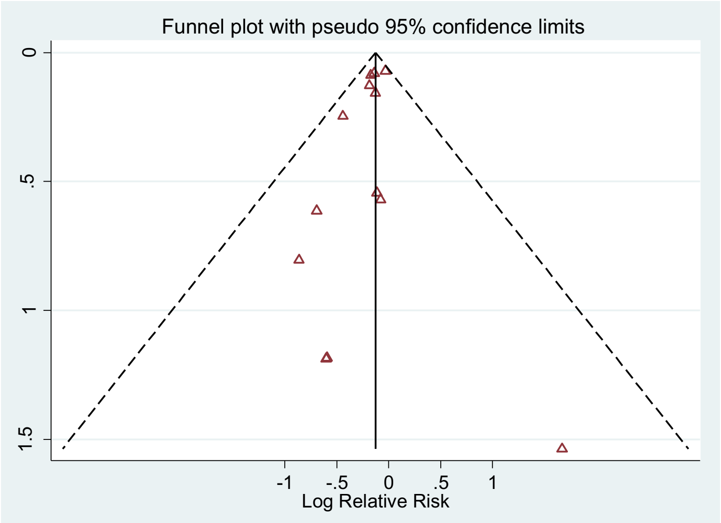

Supplementary Table 1: Study characteristics for randomized controlled trials

Author (Trial)	Arm	Year	Intervention	Control	Entry-EF cut-off	Follow-up (months)	Intervention (N)	Control (N)	Total (N)	Mean LVEF	Outcomes
**Ahmed (DIG)**[1]		2005	Digoxin	Placebo	45%	37	492	496	988	55	All-cause mortality, CV mortality, HFH
**Aronow**[2]		1997	Propranolol	No treatment	40%	12	79	79	158	57	All-cause mortality, CV mortality,
**Bergstrom (SWEDIC)**[3]		2004	Carvedilol	Placebo	45%	6	47	50	97	NR	HFH, biomarker
**Cleland (PEP-CHF)**[4]		2006	Perindopril	Placebo	40%	25.2	424	426	850	65	All-cause mortality, CV mortality, HFH, exercise time, biomarker
**Conraads**[5]		2012	Nebivolol	Placebo	45%	6	57	59	116	63	6MWD, VO_2, QOL, biomarker
**Davis (ALLHAT)**[6]	1	2008	Amlodipine	Chlorthalidone	50%	20.9	110	117	227	NR	All-cause mortality
**Davis (ALLHAT)**	2	2008	Lisinopril	Chlorthalidone		20.9	98		215	NR	
**Davis (ALLHAT)**	3	2008	Doxazosin	Chlorthalidone		18.6	79	66	145	NR	
**Deswal (RAAM-PEF)**[7]		2011	Eplenerone	Placebo	50%	6	22	22	44	62	HFH, 6MWD
**Edelmann (ALDO-DHF)**[8]		2013	Spironolactone	Placebo	50%	12	213	209	422	67	All-cause mortality, exercise time, 6MWD, VO_2, QOL, biomarker
**Hung**[9]		2003	Verapamil	Placebo	50%	3	15	15	15	70	Exercise time
**Kasama**[10]		2005	Candesartan	Placebo	40%	6	25	25	50	55	Biomarker
**Kitzman**[11]		2010	Enalapril	Placebo	50%	12	35	36	71	65	Exercise time, 6MWD, VO_2, QOL, biomarker
**Kurrelmeyer**[12]		2014	Spironolactone	Placebo	50%	6	24	24	48	63	6MWD, Biomarker
**Little**[13]		2006	Losartan	Hydrochlorothiazide	50%	6	19	21	40	67	Exercise time, VO_2,
**Mak**[14]		2009	Epleronone	Placebo	45%	12	24	20	44	63	QOL, biomarker

**Massie (I-PRESERVE)**[15]		2008	Irbesartan	Placebo	45%	49.5	2067	2061	4128	60	All-cause mortality, CV mortality, HFH, QOL, biomarker
**Parthasarathy**[16]		2009	Valsartan	Placebo	40%	3.5	70	82	152	71	Exercise time, 6MWD, VO_2, QOL, biomarker
**Pitt (TOPCAT)**[17]		2014	Spironolactone	Placebo	45%	49.5	1722	1723	3445	56	All-cause mortality, CV mortality, HFH
**Rector (I-PRESERVE)**[18]		2012	Irbesartan	Placebo	45%	56	1102	1103	2205	NR	QOL
**Redfield (RELAX)**[19]		2013	Sildenafil	Placebo	50%	6	113	103	216	60	All-cause mortality, 6MWD, VO_2, QOL, biomarker
**Solomon (CHARM-Preserved)**[20]		2004	Candesartan	Placebo	40%	36.6	1514	1509	3023	54	All-cause mortality
**Takeda**[21]		2004	Carvedilol	No treatment	45%	12	19	21	40	57	HFH, QOL, biomarker
**Van Veldhuisen (SENIORS)**[22]		2009	Nebivolol	Placebo	35%	21	320	323	643	NA	All-cause mortality, CV mortality
**Yamamoto (J-DHF)**[23]		2013	Carvedilol	Placebo	40%	38.4	120	125	245	63	All-cause mortality, CV mortality, HFH
**Yip (HK-DHF)**[24]	1	2008	Ramipril	Diuretics	45%	12	45	50	151	67	All-cause mortality, CV mortality, HFH, 6MWD, QOL, biomarker
**Yip (HK-DHF)**[24]	2	2008	Irbesartan			12	56			68	
**Yusuf (CHARM-Preserved)**[25]		2003	Candesartan	Placebo	40%	36.6	1514	1509	3023	54	CV mortality, HFH
**Zi**[26]		2003	Quinapril	Placebo	40%	6	36	38	74	59	All-cause mortality, HFH, 6MWD
**Zile**[27]		2014	Sitaxsentan		50%	6	128	64	192	61	HFH, exercise time, QOL

Supplementary Table 2: Summary of effects for all primary and secondary outcomes by stratification

Outcome	All-cause mortality	Cardiovascular mortality	Heart failure hospitalisation	Exercise capacity	6MWD	VO_2 max	MLHFQ
**Overall**	0.96<br>0.90 to 1.03	0.95<br>0.87 to 1.03	**0.88**<br>**0.81 to 0.95**	19.92<br>-1.30 to 41.14	-1.94<br>-8.66 to 4.77	-0.16<br>-0.55 to 0.24	**-1.63**<br>**-2.94 to -0.31**
**Stratified by drug class**							
**Beta-blockers**	**0.78**<br>**0.65 to 0.94**	**0.75**<br>**0.60 to 0.94**	0.67<br>0.42 to 1.07	**39.91**<br>**14.95 to 64.87**	-26.00<br>-56.97 to 4.97	-1.50<br>-3.36 to 0.36	1.00<br>-5.85 to 7.85
**RAAS blockers**	1.00<br>0.93 to 1.08	0.99<br>0.89 to 1.09	**0.90**<br>**0.82 to 0.98**	-0.69<br>-23.59 to 22.20	1.11<br>-5.45 to 7.67	-0.27<br>-0.95 to 0.41	-
**ACE inhibitors**	1.10<br>0.85 to 1.43	0.94<br>0.62 to 1.43	0.86<br>0.64 to 1.15	-	-	-	-2.77<br>-7.15 to 1.61
**ARB**	1.02<br>0.93 to 1.12	1.02<br>0.90 to 1.14	0.92<br>0.83 to 1.02	-	-	-	**-1.84**<br>**-3.55 to -0.14**
**MRA**	0.92<br>0.79 to 1.08	0.91<br>0.74 to 1.11	0.84<br>0.71 to 1.00	-	-	-	-1.39<br>-4.85 to 2.08
**Others**	0.95<br>0.78 to 1.15	1.02<br>0.77 to 1.35	0.81<br>0.64 to 1.04	52.96<br>-1.30 to 104.13	-1.94<br>-8.65 to 4.77	-0.00<br>-0.50 to 0.50	-0.85<br>-4.49 to 2.80
Stratified by follow-up duration							
**3 to 12 months**	**0.79**<br>**0.66 to 0.95**	**0.71**<br>**0.55 to 0.90**	**0.67**<br>**0.48 to 0.94**	-	-	-	-1.03<br>-2.84 to 0.78
**>12 months**	0.99<br>0.92 to 1.06	0.99<br>0.90 to 1.08	**0.90**<br>**0.82 to 0.98**	-	-	-	**-2.30**<br>**-4.22 to -0.38**
Stratified by trial LV ejection fraction threshold							
**40 to 49%**	0.96<br>0.88 to 1.03	0.95<br>0.87 to 1.03	**0.88**<br>**0.82 to 0.96**	17.03<br>-29.36 to 63.41	-	**-1.29**<br>**-2.55 to -0.03**	**-1.82**<br>**-3.37 to -0.28**
**≥50%**	0.99<br>0.74 to 1.32	-	0.51<br>0.18 to 1.48	11.33<br>-10.56 to 33.22	-	-0.03<br>-0.34 to 0.28	-1.11<br>-3.63 to 1.42
Stratified by mean LV ejection fraction							
**<60%**	0.93<br>0.82 to 1.05	0.90<br>0.78 to 1.05	**0.85**<br>**0.76 to 0.94**	-	-	-	-
**≥60%**	0.95<br>0.88 to 1.04	1.02<br>0.89 to 1.17	0.92<br>0.82 to 1.04	-	-	-	-

Data presented as risk ratios (for all-cause and cardiovascular mortality and hospitalisation outcomes) or mean difference (exercise capacity, 6MWD, VO2 max and MLHFQ), with 95% CI. 6MWD – six-minute walk distance; MLHFQ – Minnesota living with heart failure questionnaire. Only one trial with LV ejection fraction threshold ≥50% reported cardiovascular mortality.

Supplementary Table 3: Overview of changes to BNP and NT-pro BNP following treatment in included trials

Author, Trial	Year	Drug	Follow-up	Study drug N	Study drug baseline	Study drug follow-up	Control N	Control baseline	Control follow-up	Mean change difference
**BNP**										
Bergstrom, SWEDIC	2004	Carvedilol	6 months	47	28	28	50	28	42	
Kasama	2005	Candesartan	6 months	25	202	134	25	204	193	
Kitzman	2010	Enalapril	12 months	35	78	84	36	64	67	
Kurrelmeyer	2014	Spironolactone	6 months	24	139	119	24	215	167	
Mak	2009	Eplenerone	12 months	24	219	158	20	192	123	
Parthasarathy	2009	Valsartan	14 weeks	61	93	94	74	120	110	
Takeda	2004	Carvedilol	12 months	19	172	75	21	150	174	
**NT-proBNP**										
Cleland, PEP-CHF	2006	Perindopril	2.1 years	191	335		184	453		-149
Conraads, ELANDD	2012	Nebivolol	6 months	57	147	162	59	126	99	
Edelman, ALDO-DHF	2013	Spironolactone	12 months	204	179	152	196	148	165	
Massie, I-PRESERVE	2008	Irbesartan	6 months	2067	360		2061	320		-11
Redfield, RELAX	2013	Sildenafil	24 weeks	95	757		94	648		38
Yip-1, HK-DHF	2008	Ramipril	12 months	45	488	314	50	566	334	
Yip-2, HK-DHF	2008	Irbesartan	12 months	56	568	443	50	566	334	

1. Ahmed A, Rich MW, Love TE, et al. Digoxin and reduction in mortality and hospitalization in heart failure: a comprehensive post hoc analysis of the DIG trial. *European heart journal* 2006; 27(2). http://onlinelibrary.wiley.com/o/cochrane/clcentral/articles/684/CN-00561684/frame.html
http://eurheartj.oxfordjournals.org/content/ehj/27/2/178.full.pdf.
2. Aronow WS, Ahn C, Kronzon I. Effect of propranolol versus no propranolol on total mortality plus nonfatal myocardial infarction in older patients with prior myocardial infarction, congestive heart failure, and left ventricular ejection fraction > or = 40% treated with diuretics plus angiotensin-converting enzyme inhibitors. *The American journal of cardiology* 1997;80(2):207-9. [published Online First: 1997/07/15]
3. Bergström A, Andersson B, Edner M, et al. Effect of carvedilol on diastolic function in patients with diastolic heart failure and preserved systolic function. Results of the Swedish Doppler-echocardiographic study (SWEDIC). *European journal of heart failure* 2004; 6(4). http://onlinelibrary.wiley.com/o/cochrane/clcentral/articles/721/CN-00489721/frame.html
http://onlinelibrary.wiley.com/store/10.1016/j.ejheart.2004.02.003/asset/ejhf2004-02-003.pdf?v=1&t=ieha38dx&s=afd318d8893c536f26b63147556956748ee85224
http://onlinelibrary.wiley.com/store/10.1016/j.ejheart.2004.02.003/asset/ejhf2004-02-003.pdf?v=1&t=iepjl3iu&s=18b120c69a4246bb506edb4dbd69846fccb359d3.
4. Cleland JG, Tendera M, Adamus J, et al. The perindopril in elderly people with chronic heart failure (PEP-CHF) study. *European heart journal* 2006; 27(19). http://onlinelibrary.wiley.com/o/cochrane/clcentral/articles/489/CN-00576489/frame.html
http://eurheartj.oxfordjournals.org/content/ehj/27/19/2338.full.pdf.
5. Conraads VM, Metra M, Kamp O, et al. Effects of the long-term administration of nebivolol on the clinical symptoms, exercise capacity, and left ventricular function of patients with diastolic dysfunction: Results of the ELANDD study. *European Journal of Heart Failure* 2012;14(2):219-25. doi: http://dx.doi.org/10.1093/eurjhf/hfr161
6. Davis BR, Kostis JB, Simpson LM, et al. Heart failure with preserved and reduced left ventricular ejection fraction in the antihypertensive and lipid-lowering treatment to prevent heart attack trial. *Circulation* 2008;118(22):2259-67. doi: 10.1161/circulationaha.107.762229 [published Online First: 2008/11/13]
7. Deswal A, Richardson P, Bozkurt B, et al. Results of the Randomized Aldosterone Antagonism in heart failure with Preserved Ejection Fraction trial (RAAM-PEF). *Journal of Cardiac Failure* 2011;17(8):634-42. doi: http://dx.doi.org/10.1016/j.cardfail.2011.04.007
8. Edelmann F, Wachter R, Schmidt AG, et al. Effect of spironolactone on diastolic function and exercise capacity in patients with heart failure with preserved ejection fraction: The Aldo-DHF randomized controlled trial. *JAMA - Journal of the American Medical Association* 2013;309(8):781-91. doi: http://dx.doi.org/10.1001/jama.2013.905
9. Hung MJ, Cherng WJ, Kuo LT, et al. Effect of verapamil in elderly patients with left ventricular diastolic dysfunction as a cause of congestive heart failure. *International journal of clinical practice* 2002; 56(1). http://onlinelibrary.wiley.com/o/cochrane/clcentral/articles/681/CN-00377681/frame.html.
10. Kasama S, Toyama T, Kumakura H, et al. Effects of candesartan on cardiac sympathetic nerve activity in patients with congestive heart failure and preserved left ventricular

ejection fraction. *Journal of the American College of Cardiology* 2005;45(5):661-7. doi: 10.1016/j.jacc.2004.11.038 [published Online First: 2005/03/01]
11. Kitzman DW, Hundley WG, Brubaker PH, et al. A randomized double-blind trial of enalapril in older patients with heart failure and preserved ejection fraction: effects on exercise tolerance and arterial distensibility. *Circulation Heart failure* 2010; 3(4). http://onlinelibrary.wiley.com/o/cochrane/clcentral/articles/761/CN-00752761/frame.html
http://circheartfailure.ahajournals.org/content/3/4/477.full.pdf.
12. Kurrelmeyer KM, Ashton Y, Xu J, et al. Effects of spironolactone treatment in elderly women with heart failure and preserved left ventricular ejection fraction. *Journal of cardiac failure* 2014; 20(8). http://onlinelibrary.wiley.com/o/cochrane/clcentral/articles/406/CN-00998406/frame.html
http://www.onlinejcf.com/article/S1071-9164(14)00215-2/abstract.
13. Little WC, Zile MR, Klein A, et al. Effect of losartan and hydrochlorothiazide on exercise tolerance in exertional hypertension and left ventricular diastolic dysfunction. *The American journal of cardiology* 2006;98(3):383-5. doi: 10.1016/j.amjcard.2006.01.106 [published Online First: 2006/07/25]
14. Mak GJ, Ledwidge MT, Watson CJ, et al. Natural History of Markers of Collagen Turnover in Patients With Early Diastolic Dysfunction and Impact of Eplerenone. *Journal of the American College of Cardiology* 2009;54(18):1674-82. doi: http://dx.doi.org/10.1016/j.jacc.2009.08.021
15. Massie BM, Carson PE, McMurray JJ, et al. Irbesartan in patients with heart failure and preserved ejection fraction. *N Engl J Med* 2008;359(23):2456-67. doi: http://dx.doi.org/10.1056/NEJMoa0805450
16. Parthasarathy HK, Pieske B, Weisskopf M, et al. A randomized, double-blind, placebo-controlled study to determine the effects of valsartan on exercise time in patients with symptomatic heart failure with preserved ejection fraction. *European journal of heart failure* 2009; 11(10). http://onlinelibrary.wiley.com/o/cochrane/clcentral/articles/071/CN-00728071/frame.html
http://www.ncbi.nlm.nih.gov/pmc/articles/PMC2754503/pdf/hfp120.pdf.
17. Pitt B, Pfeffer MA, Assmann SF, et al. Spironolactone for heart failure with preserved ejection fraction. *The New England journal of medicine* 2014; 370(15). http://onlinelibrary.wiley.com/o/cochrane/clcentral/articles/299/CN-00989299/frame.html.
18. Rector TS, Carson PE, Anand IS, et al. Assessment of long-term effects of irbesartan on heart failure with preserved ejection fraction as measured by the minnesota living with heart failure questionnaire in the irbesartan in heart failure with preserved systolic function (I-PRESERVE) trial. *Circ* 2012;5(2):217-25. doi: http://dx.doi.org/10.1161/CIRCHEARTFAILURE.111.964221
19. Redfield MM, Chen HH, Borlaug BA, et al. Effect of phosphodiesterase-5 inhibition on exercise capacity and clinical status in heart failure with preserved ejection fraction: A randomized clinical trial. *JAMA - Journal of the American Medical Association* 2013;309(12):1268-77. doi: http://dx.doi.org/10.1001/jama.2013.2024
20. Solomon SD, Wang D, Finn P, et al. Effect of candesartan on cause-specific mortality in heart failure patients: the Candesartan in Heart failure Assessment of Reduction in Mortality and morbidity (CHARM) program. *Circulation* 2004;110(15):2180-3. doi: 10.1161/01.cir.0000144474.65922.aa [published Online First: 2004/10/07]

21. Takeda Y, Fukutomi T, Suzuki S, et al. Effects of carvedilol on plasma B-type natriuretic peptide concentration and symptoms in patients with heart failure and preserved ejection fraction. *American journal of cardiology* 2004; 94(4). http://onlinelibrary.wiley.com/o/cochrane/clcentral/articles/248/CN-00481248/frame.html
http://www.ajconline.org/article/S0002-9149(04)00707-6/abstract.
22. van Veldhuisen DJ, Cohen-Solal A, Bohm M, et al. Beta-Blockade With Nebivolol in Elderly Heart Failure Patients With Impaired and Preserved Left Ventricular Ejection Fraction. Data From SENIORS (Study of Effects of Nebivolol Intervention on Outcomes and Rehospitalization in Seniors With Heart Failure). *Journal of the American College of Cardiology* 2009;53(23):2150-58. doi: http://dx.doi.org/10.1016/j.jacc.2009.02.046
23. Yamamoto K, Origasa H, Hori M. Effects of carvedilol on heart failure with preserved ejection fraction: the Japanese Diastolic Heart Failure Study (J-DHF). *European journal of heart failure* 2013; 15(1). http://onlinelibrary.wiley.com/o/cochrane/clcentral/articles/537/CN-00859537/frame.html
http://onlinelibrary.wiley.com/store/10.1093/eurjhf/hfs141/asset/ejhfhfs141.pdf?v=1&t=iefjj60q&s=05fb43d282267b248efddc37e10927936f94060d.
24. Yip GW, Wang M, Wang T, et al. The Hong Kong diastolic heart failure study: a randomised controlled trial of diuretics, irbesartan and ramipril on quality of life, exercise capacity, left ventricular global and regional function in heart failure with a normal ejection fraction. *Heart (British Cardiac Society)* 2008; 94(5). http://onlinelibrary.wiley.com/o/cochrane/clcentral/articles/478/CN-00629478/frame.html
http://heart.bmj.com/content/94/5/573.full.pdf.
25. Yusuf S, Pfeffer MA, Swedberg K, et al. Effects of candesartan in patients with chronic heart failure and preserved left-ventricular ejection fraction: the CHARM-Preserved Trial. *Lancet (London, England)* 2003; 362(9386). http://onlinelibrary.wiley.com/o/cochrane/clcentral/articles/464/CN-00440464/frame.html
http://www.thelancet.com/journals/lancet/article/PIIS0140-6736(03)14285-7/abstract.
26. Zi M, Carmichael N, Lye M. The effect of quinapril on functional status of elderly patients with diastolic heart failure. *Cardiovascular drugs and therapy / sponsored by the International Society of Cardiovascular Pharmacotherapy* 2003; 17(2). http://onlinelibrary.wiley.com/o/cochrane/clcentral/articles/791/CN-00457791/frame.html.
27. Zile MR, Bourge RC, Redfield MM, et al. Randomized, double-blind, placebo-controlled study of sitaxsentan to improve impaired exercise tolerance in patients with heart failure and a preserved ejection fraction. *Jacc* 2014;Heart failure. 2(2):123-30. doi: http://dx.doi.org/10.1016/j.jchf.2013.12.002
